# Alzheimer’s associated amyloid and tau deposition co-localizes with a homeostatic myelin repair pathway in two mouse models of post-stroke mixed dementia

**DOI:** 10.1186/s40478-018-0603-4

**Published:** 2018-09-24

**Authors:** Thuy-Vi V. Nguyen, Megan Hayes, Jacob C. Zbesko, Jennifer B. Frye, Nicole R. Congrove, Nadia P. Belichenko, Brian S. McKay, Frank M. Longo, Kristian P. Doyle

**Affiliations:** 1 Department of Immunobiology, University of Arizona, 1656 E. Mabel Street, Tucson, AZ 85719 USA; 2 Department of Neurology, University of Arizona, 1656 E. Mabel Street, Tucson, AZ 85719 USA; 3 Arizona Center on Aging, University of Arizona, 1656 E. Mabel Street, Tucson, AZ 85719 USA; 4 Department of Ophthalmology and Vision Science, University of Arizona, Tucson, AZ USA; 5 Department of Neurology and Neurological Sciences, Stanford University, Stanford, CA USA

**Keywords:** Stroke, Alzheimer’s disease, Mixed dementia, Amyloid, Tau, BACE1, Neuregulin, Myelin repair

## Abstract

The goal of this study was to determine the chronic impact of stroke on the manifestation of Alzheimer’s disease (AD) related pathology and behavioral impairments in mice. To accomplish this goal, we used two distinct models. First, we experimentally induced ischemic stroke in aged wildtype (wt) C57BL/6 mice to determine if stroke leads to the manifestation of AD-associated pathological β-amyloid (Aβ) and tau in aged versus young adult wt mice. Second, we utilized a transgenic (Tg) mouse model of AD (hAPP-SL) to determine if stroke leads to the worsening of pre-existing AD pathology, as well as the development of pathology in brain regions not typically expressed in AD Tg mice. In the wt mice, there was delayed motor recovery and an accelerated development of cognitive deficits in aged mice compared to young adult mice following stroke. This corresponded with increased brain atrophy, increased cholinergic degeneration, and a focal increase of Aβ in areas of axonal degeneration in the ipsilateral hemisphere of the aged animals. By contrast, in the hAPP-SL mice, we found that ischemia induced aggravated behavioral deficits in conjunction with a global increase in Aβ_,_ tau, and cholinergic pathology compared to hAPP-SL mice that underwent a sham stroke procedure. With regard to a potential mechanism, in both models, we found that the stroke-induced Aβ and tau deposits co-localized with increased levels of β-secretase 1 (BACE1), along with its substrate, neuregulin 1 (NGR1) type III, both of which are proteins integral for myelin repair. Based on these findings, we propose that the chronic sequelae of stroke may be ratcheting-up a myelin repair pathway, and that the consequent increase in BACE1 could be causing an inadvertent cleavage of its alternative substrate, AβPP, resulting in greater Aβ seeding and pathogenesis.

## Introduction

People over the age of 55 have a 1 in 6 lifetime risk of suffering a stroke, and 1 in 10 people aged 65 years or older are diagnosed with Alzheimer’s disease (AD) [6, 17]. Currently, there are more than 6.5 million stroke survivors in the United States, a third of whom will develop post-stroke dementia, within 3-6 months of the ischemic event, and there are more than 5 million Americans living with AD [83]. In light of these facts, it is unsurprising that the most common form of mixed dementia is the co-morbidity of vascular pathology, such as the presence of chronic stroke infarcts, alongside AD-associated pathology. Indeed, post-mortem analyses from the National Institute of Health’s Rush Memory and Aging Project suggest that nearly 50% of clinically diagnosed AD patients have a mixture of infarct and AD (amyloid plaques and neurofibrillary tangles) pathologies [27, 80, 82]. Yet despite this evidence for the co-existence of stroke and AD-like pathology, little is known about the role that ischemic infarcts play in the pathogenesis, distribution, and burden of toxic amyloid and tau species seen in AD.

With regard to prior animal research that has examined the impact of stroke on the pathology and cognitive decline related to the AD phenotype, Qui and colleagues have shown that 2 months of cerebral hypoperfusion enhances phosphorylated (p)-tau in adult Aβ precursor protein (AβPP) transgenic (Tg) mice [70]. In addition, Lee and colleagues have demonstrated that cerebral hypoperfusion worsens cognitive dysfunction in adult AβPP Tg mice [52]. Likewise, Pimentel-Coelho and colleagues found that 6 weeks of cerebral hypoperfusion induces cognitive dysfunction in young adult AβPP/presenilin 1 (PS1) Tg mice that is strongly correlated with Aβ plaque burden, but not in their wildtype (wt) littermates [68]. However, these cerebral hypoperfusion studies do not provide information with regard to how amyloid manifests in response to the chronic consequences of a distinct infarct.

This gap in knowledge is partly filled by work from the Simpkins research group, which has shown that a middle cerebral artery occlusion (MCAO) in young adult rats results in apoptosis and tau pathology at 2 and 24 hours post-stroke, respectively [101, 102]. Similarly, Garcia-Alloza and colleagues have reported that a MCAO or photothrombotic (PT) stroke in adult and mature AβPP/PS1dE9 mice correlates with significant accumulation of Aβ plaques at 7 days post-stroke [30]. Whitehead and colleagues found that giving a vasoconstrictor, endothelin-1 (ET-1), to adult AβPP Tg mice results in a stroke with increased AβPP and tau-2 expression by 8 days post-stroke [104]. However, these studies have only investigated the effect of ischemia on the development of AD-associated amyloid, tau, AβPP proteins in the post-stroke timeframe of hours and days.

With regard to the long-term impact of stroke on the development of pathology and other phenotypic markers associated with AD, Back and colleagues recently reported that chronic cerebral hypoperfusion following MCAO in rats results in synergistic impairment of spatial memory, enhanced neuroinflammation, and aggravated amyloid pathology after 12 weeks; however, they only found a significant increase in amyloid burden and inflammation in the ipsilateral hemisphere [7]. Likewise, in another rat model, after waiting 12 weeks post-stroke, Sharp and colleagues detected the presence of Aβ in the ipsilateral cortex surrounding the infarct, but only after administering lipopolysaccharide (LPS) as an inflammatory stimulus [109]. Recently, Ong and colleagues found toxic Aβ oligomers in the thalami of adult mice at 6 weeks following PT stroke. Using rats instead of mice, they also found exacerbation of pathology, but only when the rats were exposed to chronic behavior-induced stress in addition to a PT stroke [66]. These studies, however, have only utilized wt mice and rats rather than Tg mouse models of AD, which have proven useful for revealing disease-related mechanisms.

Taken together, these studies all support the hypothesis that the genesis of AD-associated pathology is a chronic sequela of ischemic stroke under certain circumstances, and they have introduced important models of mixed dementia (i.e., the co-existence of infarct and AD-related pathologies) that can be used to help untangle causality. However, to fill a gap that still remains in the field, the goal of this study was to evaluate the impact of stroke on the development of pathology, degeneration, and behavioral dysfunction associated with the AD phenotype over a chronic time period in aged and young wildtype mice, as well as a Tg AβPP mouse line (hAPP-SL).

To accomplish this goal, we employed a distal MCAO plus hypoxia stroke model, which our research group previously demonstrated causes delayed cognitive decline [21]. Considering that AD-related pathology has been reported in the brains of patients with post-stroke dementia, this is a pertinent model for investigating the pathophysiology of post-stroke mixed dementia. However as a caveat, and to be clear, this study is not modeling mixed AD vascular dementia, associated with the presence of white matter lesions, lacunar infarcts, microinfarcts, and microbleeds [39]. Rather, this study is using mice to specifically emulate how an ischemic stroke in a large to medium size artery impacts AD related pathology in humans.

Using our model of post-stroke mixed dementia, we found that multiple neuropathological abnormalities associated with AD develop over the course of 8 weeks in the white matter tracts (mainly in the internal capsule and thalamus regions, where axonal degeneration resulting from a stroke can be found) in the ipsilateral hemisphere of aged wt mice, but not young adult mice following stroke. Aged AβPP Tg mice, however, develop exacerbated archetypal London/Swedish mutation AD pathology in both hemispheres, as well as in the ipsilateral white matter tract regions apparent in wt mice at 12 weeks following stroke. In both of these models, Aβ and tau pathology overlapped with increased levels of BACE1 and its substrate NGR1 type III, both of which are critical components of a myelin repair pathway [10, 15]. Although this is not evidence of causality, these observations are in agreement with George Bartzokis’ proposed myelin model of AD as a homeostatic response to myelin breakdown [11]. That is to say, a possible interpretation of the findings reported here is that the chronic sequelae of stroke may be ratcheting-up a neuregulin dependent myelin repair pathway, and the consequent increase in BACE1 may be causing inadvertent cleavage of AβPP, resulting in greater Aβ seeding and pathogenesis. The two mouse models of post-stroke mixed dementia introduced in this study now provide powerful tools to test this working hypothesis, as well as alternative hypotheses.

## Materials and methods

### C57BL/6 and hAPP-SL mice

**Fig. 1:**
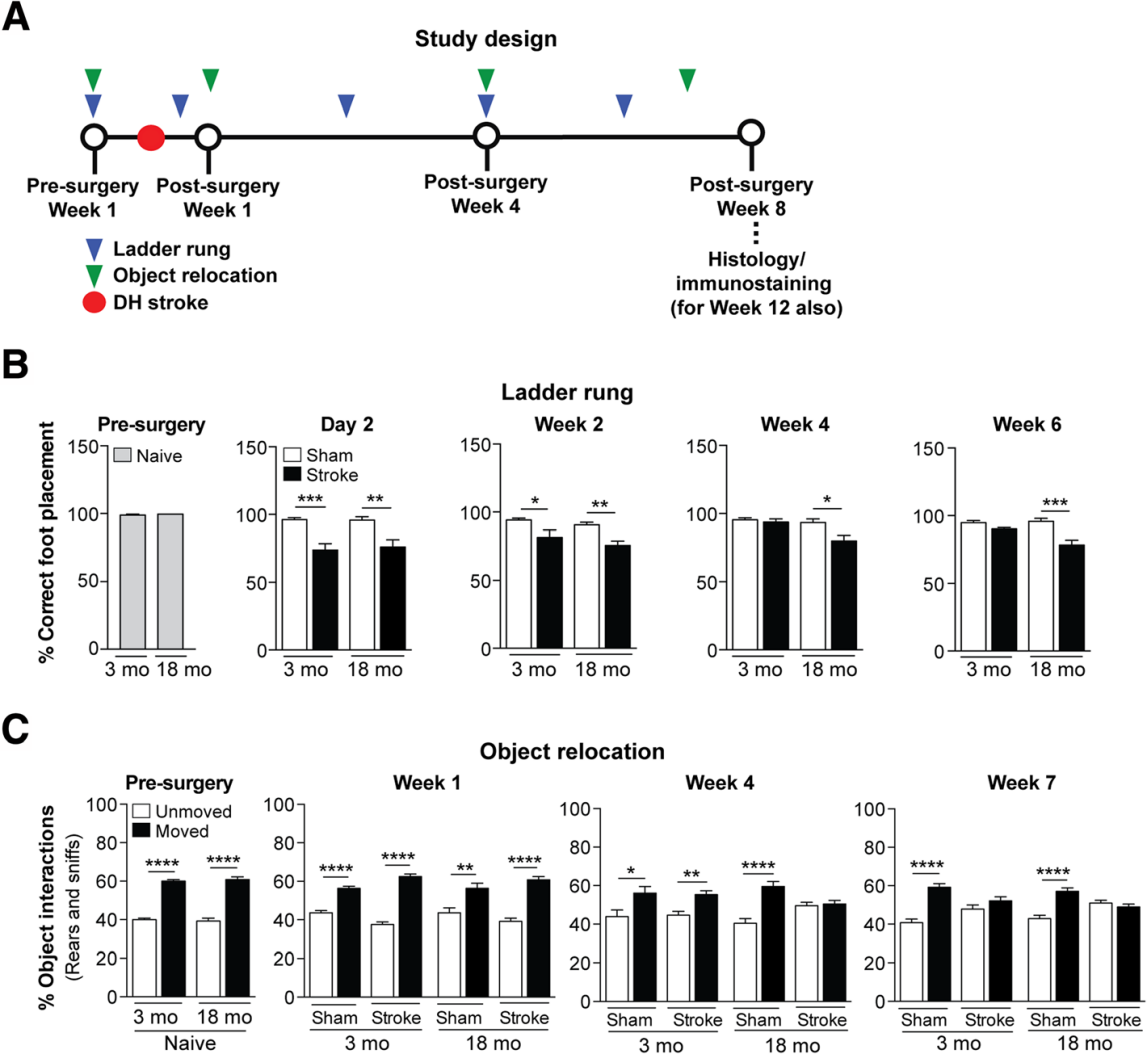
Motor recovery is impaired and there is accelerated onset of cognitive impairment in aged versus young adult C57BL/6 mice following stroke. **a** Study design: 3 and 18 month-old (mo) C57BL/6 mice were assessed on the ladder rung and object relocation tests prior to a distal hypoxic (DH) stroke or sham surgery at the indicated timepoints. Following behavioral testing, mice were euthanized and brains were harvested for histology and immunostaining at either 8 or 12 weeks post-surgery. **b** Motor ability on the ladder rung test was assessed at 1 week pre-surgery, and at 2 days, 2 weeks, 4 weeks, and 6 weeks post-surgery. At 1 week prior to a stroke, there was no difference in motor performance between 3 and 18 mo mice, as both age groups displayed a similar number of correct (nearly 100%) foot placements on the rungs. At 2 days and 2 weeks post-surgery, 3 and 18 mo stroked mice exhibited a motor deficit, as both age groups displayed a significantly fewer number of correct foot placement relative to age-matched sham mice. However, at 4 and 6 weeks post-surgery, 3 mo stroked mice exhibited motor recovery, as they displayed an indistinguishable number of correct foot placements as age-matched sham mice, which was once again nearly 100% correct. 18 mo stroked mice, however, continued to exhibit a motor deficit relative to age-matched sham mice at 4 and 6 weeks post-surgery. **c** Cognitive function using the object relocation test was assessed at 1 week pre-surgery, and at 1 week, 4 weeks, and 7 weeks post-surgery. At 1 week prior to a stroke, there was no difference in cognitive function between 3 and 18 mo mice, as both age groups displayed significantly more interactions (sniffs and rears) with moved (novel location) verses unmoved objects relative to age-matched sham mice. However, at 4 weeks post-surgery, the 18 mo stroked mice exhibited cognitive dysfunction, as they were now interacting with the two sets of objects equally, indicating that they could no longer distinguish between the moved and unmoved objects. Not until 7 weeks post-surgery did the 3 mo stroked mice exhibit cognitive dysfunction. Data represent mean ± SEM. *p<0.05, **p<0.01, ***p<0.001, and ****p<0.0001

For the purpose of investigating the chronic impact of a stroke infarct on pathogenesis related to AD in mice with no known predisposition for AD-like pathology (see Study design in Fig. 1a), we considered different ages and used both young adult and aged male C57BL/6 mice, which were purchased from The Jackson Laboratory (stock number: 000664). Each wt mouse was given a stroke or sham surgery starting at either 3 or 18 months old (mo). Behavioral testing was performed a week before surgery, and final testing completed during week 7, after which mice were sacrificed at week 8 post-surgery, when they were then 5 and 20 mo, respectively. A cohort of 17-18 mo wt mice were sacrificed at 12 weeks after surgery, when they were 20-21 mo, to extend the post-stroke time interval in order to determine the kinetics of deleterious processes that may still be progressing.

**Fig. 2:**
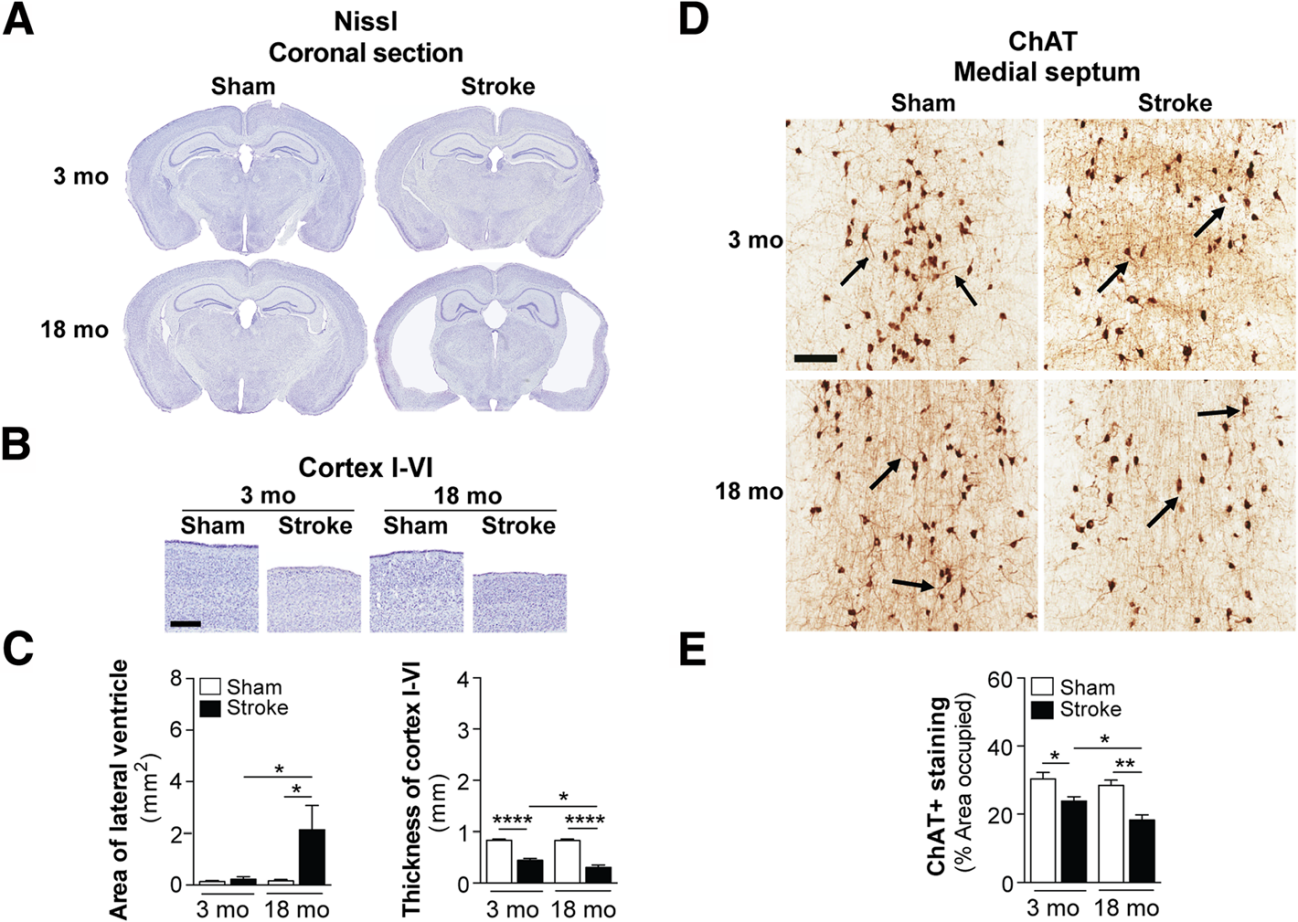
Brain atrophy and cholinergic degeneration is more pronounced in aged versus young adult C57BL/6 mice following stroke. **a** Representative 4× images of Nissl-stained whole brain sections from 3 and 18 month-old (mo), sham- and stroke-operated C57BL/6 mice at 8 weeks post-surgery. **b** Representative 2× images of Nissl-stained cortical layers I-VI of the ipsilateral primary somatosensory cortex in 3 and 18 mo, sham- and stroke-operated mice at 8 weeks post-surgery. Scale bar, 250 μm. **c** Quantification of the lateral ventricle (left graph) in the ipsilateral hemisphere revealed significant ventricle enlargement in the 18 mo stroked mice relative to the age-matched sham-operated mice. Furthermore, the 18 mo stroked mice had a significantly larger ventricle area compared to the 3 mo stroked mice. Quantification of cortical thickness (right graph) in the ipsilateral hemisphere revealed significant tissue loss or shrinkage in both the 3 and 18 mo stroked mice relative to age-matched sham-operated mice. Furthermore, the 18 mo stroked mice had significantly more tissue loss compared to the 3 mo stroke mice. **d** Representative 5× images of choline acetyltransferase (ChAT)-immunolabeled neuronal cell bodies, their neurites, and innervating projection fibers in the medial septum of the basal forebrain in 3 and 18 mo, sham- and stroke-operated C57BL/6 mice at 8 weeks post-surgery. Many neurites (arrows) in the stroked mice displayed qualitative degenerative changes, including decreased length relative to sham mice. Scale bar, 100 μm. **e** Quantification of cholinergic somas, neurites, and fibers revealed a significant reduction of ChAT+ staining in both the 3 and 18 mo stroked mice relative to the age-matched sham mice. Furthermore, the 18 mo stroked mice exhibited significantly more loss of ChAT+ staining compared to the 3 mo stroked mice. Data represent mean ± SEM. *p<0.05, **p<0.01, and ****p<0.0001

For the purpose of investigating the effects of a chronic stroke on existing AD pathology (see Study design in Fig. 2a), aged male hAPP-SL (Thy1-hAβPP^Lond/Swe^) mice were used. This transgenic mouse line 41 over-expresses human AβPP751 containing the London (V717I) and Swedish (K670M/N671L) mutations under the control of the Thy1 promoter [25, 33, 38, 47, 67, 75–77, 79], and is maintained on a C57BL/6 background. Because of a month age gap between the youngest and oldest hAPP-SL mice, they were randomized to either stroke or sham surgery groups such that there was no significant difference in age (reflective of extent of pathology) between experimental conditions. Each hAPP-SL mouse was given either a stroke or sham operation at 17-18 mo. Behavioral testing in these mice was performed a week before surgery, and final testing completed during week 11, after which mice were sacrificed 12 weeks after surgery at 20-21 mo to ensure that enough time passed to discern any enhancement in pathology due to a stroke.

All mice were housed under a 12-hour light/dark schedule with *ad libitum* access to food and water. In behavioral experiments, mice were handled by the experimenter in the procedure room for 5 days before testing, and habituated to the procedure room 2 hours prior to testing. Each behavioral testing arena and each object was thoroughly cleaned with 10% ethanol and allowed to dry between each trial. At study completion, mice were anesthetized with 5% isoflurane, followed by intracardial perfusion with 0.9% saline. Brains were extracted, and either the target regions dissected and snap frozen in liquid nitrogen for biochemical assays, or the whole brain was post-immersed in 4% paraformaldehyde and cyroprotected in 30% sucrose prior to sectioning. All behavioral testing and analyses, including biochemical and morphological analyses were performed with blinding to experimental condition, except in the case of tissue processing due to the visibility of the stroke lesion. Experiments were performed in accordance with protocols approved by the University of Arizona Institutional Animal Care and Use Committee, and were based on the National of Health Guide for the Care and Use of Laboratory Animals.

### Stroke and sham surgeries

Distal MCAO plus hypoxia (DH stroke) was performed in mice, as previously described [22, 63, 108]. Mice were anesthetized by isoflurane inhalation, and an incision was made to expose the temporalis muscle. A pocket was created in the muscle to expose the skull underneath, and the right MCA identified. A microdrill was used to expose the underlying MCA. The meninges were cut, and the vessel cauterized using a small vessel cauterizer. The wound was then closed using surgical glue. Immediately following surgery, mice were placed in a large chamber containing 9% oxygen and 91% nitrogen for 45 minutes. Sham mice underwent the same surgical steps, except for cauterization of the MCA, and were also given 9% oxygen and 91% nitrogen for 45 minutes immediately after surgery. Core body temperature was maintained at 37°C throughout surgery and hypoxia.

This stroke model creates a large infarct comprising approximately 25% of the ipsilateral hemisphere, and is adjacent to the hippocampus, but does not incorporate it to avoid an immediate memory deficit. This model has little variability, and excellent long-term survivability [22]. Hypoxia is necessary with this model because C57BL/6 mice that underdo distal MCAO without hypoxia have significantly smaller infarcts [22]. Although hypoxia is required for DH stroke, we found that other stroke paradigms such as PT stroke, which does not require hypoxia, or BALB/c mice that undergo distal MCAO without hypoxia, have similar infarct size and degree of morphological and biochemical outcomes in similar regions of the brain as C57BL/6 mice given DH stroke [22, 23, 63, 108]. Furthermore, we found that naïve mice do not differ from sham mice that undergo a sham procedure and hypoxia on behavioral, morphological, and biochemical parameters at 12 weeks post-surgery.

### Ladder rung test

The ladder rung test [69] was adapted from the ladder rung walking task [26, 59]. The apparatus consisted of two plexiglass walls (0.64 cm width × 76.20 cm length × 15.24 cm height) spaced 3.18 cm apart, just wide enough for the animal to pass through. Plexiglass rungs (10.16 cm long with a 0.32 cm diameter) were inserted across the length of the walls and spaced at a constant distance of 0.64 cm apart. Both ends of the apparatus were placed atop standard mouse housing cages so that the rungs are 38 cm from the tabletop. A desk lamp illuminating the start zone incentivized the animals to traverse the ladder, and a small igloo leading to the home cage was placed at the end zone.

The test was performed at 1-week pre-surgery for all mice, at 1, 4, and 7 weeks post-surgery for C57BL/6 mice (n=8-10 per experimental group), and at 1, 6, and 11 weeks post-surgery for hAPP-SL mice (n=16-17 per experimental group). Prior to pre-surgery testing, training was necessary to ensure that the animals were able to spontaneously traverse the apparatus. Eight training trials were required for animals to make an acceptable minimum number of limb placing errors (<12% error). Traversing animals were recorded from below the ladder with a handheld video recorder. Each animal was tested once for a given day, and each trial was analyzed frame-by-frame using standard film play-back software (VLC media player for Mac OSX). Tallying began once the first visible full gait cycle had been completed (i.e., all four limbs had been placed). If a limb was placed on a rung and was not subsequently removed, a correct step was recorded. If a limb was placed and subsequently slipped off the rung, a missed step was recorded. If a limb was placed and subsequently replaced on the same rung, a correction step was recorded. Tallying was complete after the animal’s final gait cycle. The percent correct foot placement was calculated as 100 × [correct step/(correct step + missed step + correction step)].

### Object relocation (OR) test

C57BL/6 mice (n=10-16 per experimental group) were habituated for 5 minutes to an empty arena (50 cm width × 50 cm length × 50 cm height) composed of beige acrylonitrile butadiene styrene (ABS) plastic, and containing spatial cues on two of the walls. Mice were then returned to their home cage while four different objects were placed in the arena. The objects consisted of children’s building blocks of different shapes and colors, and each was placed in a corner of the arena, 5 cm from the walls. Baseline studies showed no innate preference between objects (data not shown). Mice were allowed to explore the objects for 5 minutes and were then returned to their home cage. The positions of two of the objects were then switched. Rodents have a natural tendency to explore novelty, and in this test, spend more time exploring or interacting with objects that have been relocated. After 5 minutes, mice were then returned to the arena, and the number of interactions (rears and sniffs) for moved and unmoved objects was recorded for 5 minutes. Different sets of objects were used for each timepoint to control for biases from previously exposed objects.

### Y-maze spontaneous alternation behavior (SAB) test

hAPP-SL mice (n=16-17 per experimental group) were tested in a Y-shaped maze composed of beige ABS plastic, and consisted of two symmetrical arms and one longer arm set at a 120° angle from each other (equal arms: 7.5 cm width × 37.0 cm length × 12.5 cm height; longer arm: 7.5 cm width × 42.0 cm length × 12.5 cm height). Mice were placed at the end of the longest arm of the maze, facing outward, and allowed to freely explore the three arms for 5 minutes. Over the course of multiple entries, mice normally exhibited a tendency to visit new arms of the maze rather than visiting a recently visited arm. An entry was recorded when all four limbs of the mouse were within an arm. The number of arm entries and the number of triads were recorded by an ANY-maze behavioral video tracking software (Stoelting), and the percentage of SAB was calculated by the software.

### Novel object recognition (NOR) test

On day 1, hAPP-SL mice (n=14-16 per experimental group) were placed with cage mates for 15 minutes in the testing apparatus, a white opaque arena (38 cm width × 48 cm length × 20 cm height) made of ABS plastic. On day 2, each mouse was allowed to explore the arena alone for 15 minutes. On day 3 (object exposure and testing), each mouse was placed in the arena along with two identical objects located at different corners of the arena 5 cm from the walls. The objects consisted of children’s building blocks of different shapes and colors. Baseline studies showed no innate preference between objects (data not shown). Mice were allowed to explore the objects until 5 minutes of exploration had accrued. Object exploration was defined as contact with the object by the mouse’s nose within 2 cm of the object, which was recorded by ANY-maze behavioral video tracking software. An object recognition test lasting for 5 minutes was performed 4 hours later. For testing, mice were placed back in the box with a “familiar” object (FO), which they had previously explored earlier in the day, and a novel object (NO). The object role (novel versus familiar) and position (left versus right) were balanced within each experimental group. Different sets of objects were used for each timepoint to control for biases from previously exposed objects. The percentage of recognition index was calculated as 100 × [(NO Time)/(NO Time + FO Time)] using total time spent exploring each object computed by the software.

### Light dark transition (LDT) test

The apparatus used for the LDT test consisted of an arena (40 cm width × 40 cm length × 35 cm height) divided into two equally sized chambers (each 20 cm width × 40 cm length × 35 cm height) with an opening (5 cm × 5 cm) between them. One chamber was made of Plexiglas that was transparent, opened on top, and brightly illuminated (390 lux) overhead. The other chamber was made of black ABS plastic, enclosed with a top lid, and kept dark (2 lux). hAPP-SL mice (*n*=17 per experimental group) were each placed in the enclosed, dark chamber of the arena at the beginning of the 10-minute trial, while being tracked by ANY-maze behavioral video tracking software once it entered the transparent, light chamber by placement of the center of its body in the doorway. The time spent in the light chamber was recorded and reported by the software.

### Body weight, spleen weight, and frailty score

Physiological parameters of body and spleen weights were taken from each animal. For body weight, hAPP-SL mice (n=15-17 per experimental group) were each weighed at the pre-surgery (baseline timepoint), and at 1 week (early timepoint), 6 weeks (mid-timepoints), and 11 weeks (final timepoints) post-surgery. Spleen weights, however, were taken from mice (n=16 per experimental group) following tissue harvest at the end of the study, at 12 weeks post-surgery.

Frailty was assessed using a noninvasive scoring system developed by Whitehead and colleagues using aged C57BL/6 mice [103], in which an overall clinical frailty index was calculated for each animal based on scores across frailty parameters, age, and clinical comparisons to humans. Frailty in hAPP-SL mice (n=16-17 per experimental group) was assessed at the pre-surgery and post-surgery timepoints.

### Nissl staining

Mounted, dehydrated brain sections were cleared in xylenes, rehydrated, immersed in 0.5% cresyl violet acetate (Millipore Sigma) solution for 1 minute, rinsed with distilled water, differentiated in 0.25% acetic alcohol, dehydrated though a graded ethanol series, cleared, and coverslipped with mounting media.

### Immunostaining

Frozen coronal sections (40 μm) were taken through the entire brain using a freezing Microm HM 450 sliding microtome (ThermoFisher Scientific), and immunostained according to standard protocols [64]. The following antibodies were used: rabbit anti-Aβ_42_ (abcam; RRID: AB_443253), mouse anti-AT8 [recognizes tau phosphorylated at serine 202 and threonine 205 (p-tau^Ser202/Thr205^, ThermoFisher Scientific; RRID: AB_223647)], rabbit anti-tau (abcam, Cat No: ab32057), goat anti-choline acetyl transferase (ChAT, Millipore Sigma; RRID: AB94647), rabbit anti-BACE1 (Cell Signaling; AB_1903900), and rabbit anti-NRG1 type III (abcam, Cat No: ab23248). Briefly, free floating sections were immunolabeled with antibodies (1:300 for Aβ_42_, 1:1500 for AT8, 1:800 for ChAT, 1:400 for BACE1, and 1:200 for NRG1 type III) in conjunction with M.O.M (for AT8), ABC Vector Elite, and 3,3’-diaminobenzidine or Vector VIP kits (for AT8) (Vector Laboratories) for visualization. For immunofluorescence staining, rabbit anti-NRG1 type III (1:50), mouse anti-AT8 (1:100), chicken anti-glial fibrillary acidic protein (GFAP, 1:500, Millipore Sigma; RRID: AB_177521), and chicken anti-ionized calcium binding adaptor molecule 1 (Iba1, 1:100, abcam; RRID: AB_2728648) antibodies were used, followed by goat anti-rabbit and donkey anti-chicken Alexa Fluor secondary antibodies (Molecular Probes), or a DyLight Fluor fluorescent dye (ThermoFisher Scientific) was used following secondary antibody incubation using the M.O.M kit for AT8. Images of sections co-stained with immunofluorescence antibodies were captured with a Keyence BZ-X700 fluorescence microscope using 488 nm and 647 nm filters. Amyloid plaques were stained with 1% Thioflavin S (ThioS; Millipore Sigma), and viewed under a fluorescence microscope using the 488 filter.

### Fluoro-Jade staining and analysis

To identify the area of axonal degeneration following DH stroke, brain sections were pre-mounted on slides, air-dried, and subjected to Fluoro-Jade staining. For consistency to the methodology of measuring white matter tracts labeled with Aβ_42_, p-tau, BACE1, and NRG1 type III, one section per mouse (n=4 per experimental group) at bregma -1.70 was also analyzed for Fluoro-Jade staining. This section allows one 10× field per section (landmark: starting at the reticular thalamus nucleus) to be taken for each of the following white matter tracts in a hemisphere. Briefly, the sections were immersed in a 1% NaOH and 80% ethanol solution for 5 minutes, followed by 2 minutes each in 70% ethanol and distilled water. The sections were then transferred to a solution of 0.06% potassium permanganate (Sigma-Aldrich) for 10 minutes and rinsed in distilled water for 2 minutes. The sections were then immersed to a 0.0001% solution of Fluoro-Jade C (Biosensis) dissolved in 0.1% acetic acid (pH 3.5) for 10 minutes, washed with distilled water 3 times for 1 minute each, and then left to dry overnight at room temperature in darkness and coverslipped with Entellan (Electron Microscopy Sciences). Fluoro-Jade sections were viewed under a Keyence BZ-X700 fluorescence microscope using a 488 nm filter, and digital images captured. Fluoro-Jade fluorescent staining was measured from the digital images using histogram thresholding with NIH ImageJ analysis software and computed. The threshold was set manually to identify dense immunostaining that was distinct from the background. Values for each field within a given mouse were averaged to yield one value per mouse. The Fluoro-Jade+ staining area was expressed as a percentage of the total area analyzed.

### Western blotting

To authenticate the specificity of BACE1 and NRG1 type III antibodies, white matter regions (thalamus/internal capsule) were dissected from stroked mouse brains and sonicated in ice-cold 0.1 M phosphate buffered saline (PBS) containing 1% triton X-100 and 0.1% sodium deoxycholate, Protease Inhibitor Cocktail (1:100; Millipore Sigma), and Phosphatase Inhibitor Cocktail 2 (1:100; Millipore Sigma). Following centrifugation, the total protein concentration of each supernatant was measured using a Direct Detect Infrared Spectrometer (Millipore Sigma). Samples were then resolved by electrophoresis, transferred to a nitrocellulose membrane, and probed with the same BACE1 or NRG1 type III antibodies used for immunostaining throughout the manuscript: rabbit anti-BACE1 (1:1000, Cell Signaling) and rabbit anti-NRG1 type III (1:1000, Abcam). Following incubation with the appropriate secondary antibodies, proteins were visualized using a chemiluminescence detection system (GE Healthcare Life Sciences).

### Anti-Aβ_42_ antisera affinity subtraction

Affinity chromatography was used to verify specificity of the anti-Aβ_42_ antibody produced against the Aβ_1-42_ peptide (rat/mouse form, abcam, Cat No: ab120959). Briefly, the immunizing peptide was immobilized on aldehyde-activated agarose beads (AminoLink Plus Coupling Resin, ThermoFisher Scientific), as per the manufacturer’s directions. After coupling the peptide covalently to the immobilized support, the column was washed extensively in quenching buffer to block any remaining active sites. Subsequently, the column was flushed with 10 mL of 0.1 M PBS. To remove antibodies against Aβ_42_ from the antisera, the sera was passed over the resin bed subtracting antibodies with affinity to the Aβ_1-42_ peptide. We performed parallel immunostaining using the complete antisera and the Aβ_1-42_ peptide subtracted antisera as a negative control. There was no staining detected using the peptide subtracted sera, indicating the specificity of staining achieved with the complete sera.

### Quantitation of brain atrophy

Two coronal brain sections (containing the hippocampi) per mouse (n=4-5 per experimental group) at bregma -1.70 and -1.94 based on the mouse brain atlas of Franklin and Paxinos 3^rd^ edition [28] were analyzed for anatomical abnormalities via Nissl staining. The area of the lateral ventricles, and the thickness of the primary somatosensory cortex (landmarks: from the corpus callosum through cortical layers I-VI) of each hemisphere per section were manually traced using ImageJ (National Institute of Health) analysis software. The area of the ventricles and the thickness of the cortex were then computed by ImageJ. For each mouse, the values from each section were averaged to yield one value for each measurement (area of the ventricle or thickness of the cortex) per mouse.

### Quantitation of cholinergic degeneration

ChAT-immunolabeled cholinergic somas and their neurites in the medial septum of the basal forebrain, which innervate hippocampal and cortical areas, as well as innervating fibers into the medial septum, were assessed in two sections per mouse (n=4-8 mice per experimental group). To generate an image of the complete medial septal nucleus in each section, four 10× fields were taken throughout the medial septum between bregma +1.18 and +0.74 per section, and stitched together to generate the entire medial septum for quantification. ChAT+ staining was measured from the digital images using histogram thresholding with NIH ImageJ analysis software and computed. The threshold was set manually to identify dense immunostaining that was distinct from the background. Values for each section within a given mouse were averaged to yield one value per mouse. The immunostained area was expressed as a percentage of the total area analyzed.

For assessment of dystrophic cholinergic neurites in the cortex, 5-6 sections per mouse (n=7-9 per experimental group) were analyzed. Two non-overlapping, adjacent 10× fields of the cortex comprising the cingulate, motor, and primary somatosensory areas in each hemisphere between bregma +1.18 and -1.94 were analyzed, for a total of 10-12 fields per mouse. ChAT+ staining of dystrophic neurites was measured from the digital images using histogram thresholding with NIH ImageJ analysis software and computed. The threshold was set manually to identify dense immunostaining that was distinct from the background. Values for each field within a given mouse were averaged to yield one value per mouse. The immunostained area was expressed as a percentage of the total area analyzed.

### Quantitation of Aβ_42_ immunoreactivity

In C57BL/6 mice (n=4-8 per experimental group), one section per mouse containing regions of white matter tracts following DH stroke surgery at bregma -1.70 were analyzed. This section allows two to three non-overlapping, adjacent 10× fields to be taken for each of the following white matter tracts in each hemisphere: (i) 2 fields for the internal capsule region (landmarks: in the upper part bordered by the stria terminalis); (ii) 2 fields for the thalamus regions (landmarks: at the reticular thalamus nucleus to the ventrolateral thalamic nucleus); and (iii) 3 fields for the corpus callosum region (landmarks: bordered by the indusium griseum and the dorsal fornix). Aβ+ immunoreactivity was measured from the digital images using histogram thresholding with NIH ImageJ analysis software and computed. The threshold was set manually to identify dense immunostaining that was distinct from the background. Values for each field within a given mouse were averaged to yield one value per mouse. The immunostained area was expressed as a percentage of the total area analyzed.

For consistency, one section per mouse at bregma -1.70 was also analyzed for Aβ_42_ immunoreactivity in the hAPP-SL mice (n=6-8 per experimental group). However, both white matter tracts as well as grey matter regions in these mice were quantified due to the much more widespread deposition of Aβ_42_ following stroke or sham surgery. Therefore, two to three non-overlapping, adjacent 10× fields were taken from each hemisphere: (i) 3 fields of the cortex (landmarks: beginning at the retrosplenial granular/dysgranular to the primary/secondary somatosensory areas); (ii) 2 fields of the central part of the hippocampus (landmarks: comprising the oriens layer into the dentate gyrus to CA1 to CA2/3; (iii) 2 fields for the internal capsule region (landmarks: in the upper part bordered by the stria terminalis); and (iv) 2 fields for the thalamus (landmarks: at the reticular thalamus nucleus to the ventrolateral thalamic nucleus). Aβ+ immunoreactivity was measured from the digital images using histogram thresholding with NIH ImageJ analysis software and computed. The threshold was set manually to identify dense immunostaining that was distinct from the background. Values for each field within a given mouse were averaged to yield one value per mouse. The immunostained area was expressed as a percentage of the total area analyzed.

In the same hAPP-SL mice, 3 sections per mouse (n=7-8 per experimental group) between bregma -1.34 and -2.18 were also analyzed for ThioS-stained fibrillary Aβ. 4× fields were imaged from each brain section. The percentage occupied by ThioS+ staining was computed using NIH ImageJ software and computed. Values for each section per mouse were averaged to yield one value per mouse. The area of ThioS+ staining was expressed as a percentage of the total area analyzed.

### Quantitation of tau immunoreactivity

For assessment of AT8 (p-tau) immunoreactivity, sections in regions encompassing areas of white matter tracts following DH stroke surgery between bregma -1.46 and -2.18 were analyzed (n=7-9 per experimental group). For each of the following regions, two to three non-overlapping, adjacent 10× fields were taken for each section from each hemisphere: (i) Cortex: 3 sections per mouse, 3 fields per section (landmark: at the retrosplenial granular/dysgranular to the primary/secondary somatosensory areas); (ii) Internal capsule: 3 sections per mouse, 1 field per section (landmark: starting at the upper part bordered by the stria terminalis); and (iii) Thalamus: 1 section per mouse, 1 field per section (landmark: starting at the reticular thalamus nucleus). p-tau+ staining was quantified from the digital images using histogram thresholding with NIH ImageJ analysis software and computed. The threshold was set manually to identify dense immunostaining that was distinct from the background. The immunostained area was expressed as the percentage of the total area analyzed.

### Quantitation of BACE1 and NRG1 type III immunoreactivity

Similar to quantification of tau immunoreactivity, for assessment of BACE1-immunolabeled dystrophic neurites, sections in regions encompassing areas of white matter tracts following DH stroke surgery between bregma -1.46 and -2.18 were analyzed (n=6-11 per experimental group). Briefly, for each of the following regions, two to three non-overlapping, adjacent 10× fields were taken for each section from each hemisphere: (i) Cortex: 3 sections per mouse, 3 fields per section (landmark: at the retrosplenial granular/dysgranular to the primary/secondary somatosensory areas); (ii) CA1 region of the hippocampus: 2-3 sections per mouse, 2-3 fields per section (landmark: from the oriens layer to the radiatum layer); and (iii) Thalamus: 3 sections per mouse, 1 field per section (landmark: at the reticular thalamus nucleus). BACE1+ and NRG1 type III+ staining was quantified from the digital images using histogram thresholding with NIH ImageJ analysis software and computed. The threshold was set manually to identify dense immunostaining that was distinct from the background. The immunostained area was expressed as the percentage of the total area analyzed.

### Multiplex immunoassays

For mouse brains, the cortex (excluding the stroke lesion) and white matter regions (thalamus/internal capsule) were dissected. Following dissections, mouse brain samples were snap frozen in liquid nitrogen. Tissue samples were then sonicated in ice-cold 0.1 M PBS containing 1% triton X-100 and 0.1% sodium deoxycholate, Protease Inhibitor Cocktail, and Phosphatase Inhibitor Cocktail 2. Following centrifugation, the total protein concentration of each supernatant was measured using a Direct Detect Infrared Spectrometer. Total soluble Aβ_42_ in mouse brain regions were then quantified using a customized mouse single-plex magnetic bead assay kit (Millipore Sigma), according to the manufacturer’s recommendations and protocols. Each lysate sample, standard, and quality control were measured in duplicate. Plates were read using a MAGPIX instrument platform (Luminex), and results were analyzed using MILLIPLEX Analyst 5.1 software (Millipore Sigma).

### Statistics

Data were presented as mean ± SEM. To determine normality, the Shapiro-Wilk and the Kolmogorov-Smirnov normality tests were performed. After the dataset passed the normality test, then when analyzing data consisting of the interaction of age (young adult versus aged) and experimental condition (stroke versus sham), a two-way ANOVA, followed by post-hoc Newman-Keuls testing (with the exception of brain atrophy analysis, which employed the Fisher’s LSD as the post-hoc test) was performed. For planned comparisons with explicit predictions as to the direction of difference, an ANOVA, followed by a one-tailed t-test was used. All other analyses were evaluated using an ANOVA and a two-tailed t-test. In the case that the dataset did not pass the normality test, a Mann-Whitney test or an ANOVA, followed by a Dunnett’s post-hoc test was used. Significance was set at *p*<0.05. For all figures, *p is <0.05, **p is <0.01, ***p is <0.001, and ****p is <0.0001.

## Results

### Motor recovery is impaired and there is accelerated onset of cognitive impairment in aged versus young adult C57BL/6 mice following stroke

Most stroke, AD, and mixed dementia patients are 65 years of age or older [4, 46]. Therefore, in the first part of this study, we examined the impact of age on the development of AD-associated pathological markers following a stroke in wt mice. To accomplish this goal, we induced a DH stroke or sham surgery in young adult (3 mo) and aged (18 mo) C57BL/6 mice and compared behavioral outcomes over the course of 8 weeks, and neuropathological outcomes at the end of 8 weeks (see Study design in Fig. 1a). To measure motor recovery, we performed an assessment of spontaneous gait using the horizontal ladder rung test. This test measures limb placement errors on a ladder [22], and previously, we showed that following DH stroke, C57BL/6 mice at 5 months of age display a significant motor impairment of the front limb contralateral to the stroke starting at day 1, which continues until week 2 post-surgery [22]. At baseline or pre-surgery testing, we found no difference in the ability of the 3 and 18 mo mice to traverse the ladder (Fig. 1b). However, as expected, on day 2 following a stroke, performance was significantly impaired on the ladder, which localized to the contralateral (left) front limb in both age groups.

This motor deficit persisted in both the 3 and 18 mo mice into the second week following a stroke. However, between 2 and 4 weeks post-surgery, there was a significant recovery of ladder traversing ability in the 3 mo mice, with the stroked mice performing at an equivalent level to their age-matched sham-operated counterparts at 4 and 6 weeks post-surgery. This recovery of limb function did not occur in the 18 mo stroked mice. The 18 mo stroked mice continued to perform significantly worse than their aged-matched sham-operated counterparts for the duration of the study. These data indicate that motor recovery following a stroke is impaired in aged compared to young adult C57BL/6 mice.

Next, we assessed cognitive status through the use of the OR test. We previously reported that DH stroke in 3-5 mo C57BL/6 mice results in impairment of hippocampal function at 7 weeks post-surgery, as assessed by both the Y-maze SAB and OR tests [23]. Here, we employed the OR test to determine how age in wt mice affects the chronic impact of a stroke on a task of short-term spatial memory [53, 111]. As seen in Fig. 1c, prior to surgery, the 3 and 18 mo mice were equally able to distinguish between a set of moved and unmoved objects by spending significantly more time interacting with the relocated objects. At 1 week post-surgery, stroke- and sham-operated mice from both age groups continued to be able to distinguish between the moved and unmoved objects. However, at 4 weeks post-surgery, though the 3 mo stroke- and sham-operated mice, and 18 mo sham-operated mice were able to distinguish between the moved and unmoved objects, suggesting intact short-term spatial memory, the 18 mo stroked mice were unable to recognize which objects were relocated. An equivalent, but delayed cognitive deficit subsequently appeared at 7 weeks post-surgery in the 3 mo stroked mice. These data indicate that stroke induces an accelerated onset of cognitive impairment in aged compared to young adult wt mice.

### Brain atrophy is more pronounced in the ipsilateral hemisphere of aged versus young adult C57BL/6 mice following stroke

Enlargement of the lateral ventricles and cortical shrinkage appear in both human [5, 58] and animal models [1, 34, 73, 112] of dementia. Atrophy of the brain likely results from progressive degeneration of neurons, and the loss of these neurons is a likely contributor to the manifestation of dementia. Previously, we demonstrated that DH stroke causes delayed ipsilateral cortical atrophy in 3-5 mo C57BL/6 mice at 8 weeks post-surgery, and that atrophy is correlated with a loss of neuronal specific nuclear protein (NeuN)+ cells in the peri-infarct cortex and external capsule [108]. Here, we investigated using Nissl-staining, whether the severe behavioral deficits present in aged wt stroked mice (Fig. 1b and c) were associated with more severe brain atrophy than occurs in 3 mo mice. This analysis revealed no significant difference in the area of the ipsilateral lateral ventricle of the 3 mo stroked mice compared to their aged-matched sham operated counterparts at 8 weeks post-surgery (Fig. 2a and c). However, there was a significant increase in the area of the ipsilateral lateral ventricle of the 18 mo stroked mice compared to their 18 mo sham operated counterparts (Fig. 2a and c). Furthermore, although there was a significant difference in cortical tissue loss, or thinning, of the ipsilateral hemisphere in stroke- compared to sham-operated mice of both age groups, the 18 mo stroked mice exhibited significantly more tissue loss than the 3 mo stroked mice (Fig. 2b and c). These data demonstrate that brain atrophy is more pronounced in the ipsilateral hemisphere of aged versus young adult C57BL/6 mice following stroke.

### There is increased cholinergic degeneration in aged versus young adult C57BL/6 mice following stroke

The dysfunction and loss of cholinergic neurons and their projections are among the earliest pathological events in age-related dementias such as AD and vascular dementia. Cholinergic neurons primarily originate in the basal forebrain, though they are found in lower density in regions such as the cortex and striatum. They provide the main source of acetylcholine to the cortex and hippocampus through their projection fibers, and as a result, are critical for attention, cognition, and psychological well-being [18, 24, 65, 99, 105]. Previously, we observed loss of cholinergic neurons in the ipsilateral cortex of 3-5 mo C57BL/6 mice at 7 weeks post-stroke [108]. Here, we also found significant loss of ChAT+ cells in the medial septum of the basal forebrain of stroke- and sham-operated mice from both age groups at 8 weeks post-surgery (Fig. 2d and e). However, the 18 mo stroked mice had significantly more cholinergic loss than the 3 mo stroked mice.

### Stroke induces accumulation of Aβ and tau in white matter tracts of the ipsilateral hemisphere in aged versus young adult C57BL/6 mice

As previously mentioned, approximately 50% of clinically diagnosed AD patients are verified at post-mortem to have mixed pathology, most commonly infarct and Aβ and tau accumulation [27, 80, 82]. Therefore, we next surveyed Aβ and tau levels in the 3 and 18 mo C57BL/6 mice that had undergone stroke or sham surgery.

**Fig. 3:**
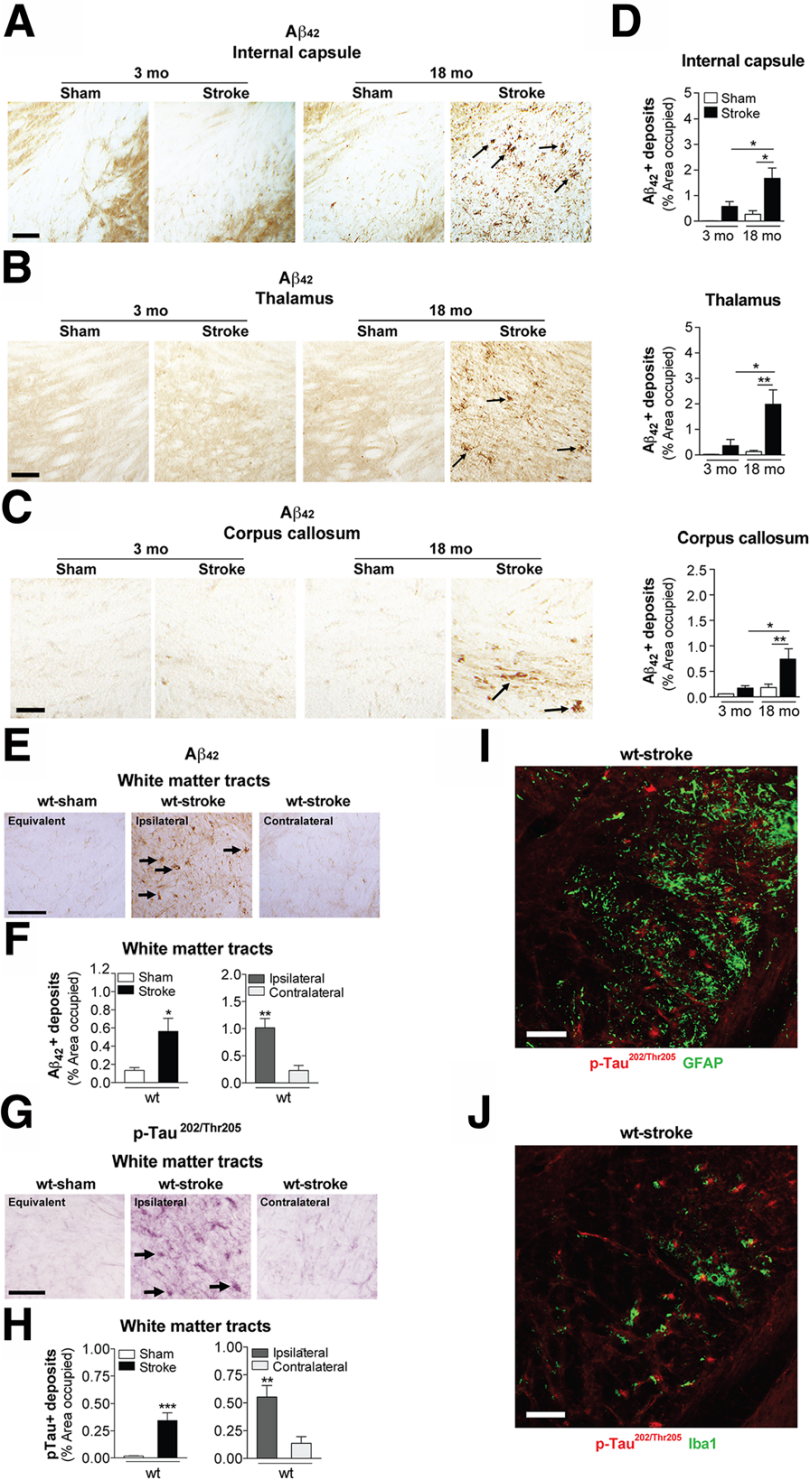
Stroke causes β-amyloid (Aβ) and phosphorylated (p) tau deposition in the white matter tracts of aged wildtype (wt) mice compared to young adult mice. Representative 10× images of Aβ_42_-immunolabeled deposits (arrows) in the white matter tracts of the (**a**) internal capsule, **b** thalamus, and (**c**) corpus callosum of the 3 and 18 mo, sham- and stroke-operated C57BL/6 mice at 8 weeks post-surgery. Scale bar, 100 μm (internal capsule and thalamus), 50 μm (corpus callosum). Nissl-stained sections to the left of each image delineate where representative images were taken. **d** Quantification of the ipsilateral hemisphere revealed a significant deposition of Aβ_42_ in the internal capsule (top graph), thalamus (middle graph), and corpus callosum (bottom graph) of the 18 mo stroked mice relative to the age-matched sham-operated mice. Furthermore, the 18 mo stroked mice had significantly more Aβ_42_ accumulation in three of the brain regions analyzed compared to the 3 mo stroked mice. **e-h** Representative 10× images of (**e**) Aβ_42_- and (**g**) p-tau-immunolabeled deposits (arrows) in white matter tracts (thalamus-internal capsule) of the 18 mo mice at 12 weeks after sham or stroke surgery (Equivalent = area imaged in wt-sham mice that is equivalent to the ipsilateral hemisphere imaged in wt-stroke mice; Contralateral = area imaged in the contralateral hemisphere of wt-stroke mice that is equivalent to the ipsilateral hemisphere of wt-stroke mice). Scale bar, 125 μm (Aβ_42_ and p-tau). Quantification of the ipsilateral and contralateral hemispheres revealed significantly more deposits of (**f**) Aβ_42_ and (**h**) p-tau in the white matter tracts of the 18 mo stroked mice compared to the age-matched sham-operated mice. Furthermore, there was also significantly more Aβ_42_ and p-tau accumulation in the white matter tracts of the ipsilateral versus the contralateral hemisphere. No Aβ_42_ signal was detected in (**i**) astrocytes (GFAP, green; n=3 mice/experimental group) or (**j**) microglia (Iba1, green; n=3 mice/experimental group). Scale bar, 125 μm. Data represent mean ± SEM. *p<0.05, **p<0.01, and ***p<0.001

As depicted in Fig. 3a-c, toxic Aβ_42_ was not significantly detected in either the 3 mo mice that underwent stroke or sham surgery, or the 18 mo mice that underwent sham surgery. However, significant accumulation of Aβ_42_ was detected in the 18 mo stroked mice at 8 weeks post-surgery. In these mice, Aβ_42_ appeared in the white matter tracts of the ipsilateral hemisphere, including the internal capsule (Fig. 3a), thalamus (Fig. 3b), and corpus callosum (Fig. 3c). Notably, quantitation revealed that there was a non-significant trend towards more Aβ_42_ deposits in the 3 mo stroked mice compared to their aged-matched sham counterparts, however, there was significantly more Aβ_42_ accumulation in the 18 mo stroked mice compared to the 3 mo stroked mice for every brain region quantified (Fig. 3d). The specificity of the Aβ_42_ antibody was confirmed by pre-absorbing the antibody with its target antigen, the Aβ_1-42_ peptide. There was no Aβ_42_+ staining detected in the pre-absorbed immunostaining sections of 18 mo stroked mice. These findings suggest that stroke alone can cause some of the abnormalities associated with AD, and that age exacerbates the manifestation of post-stroke AD-related pathological markers, such as Aβ_42_ in wt mice.

Next, to understand the kinetics of deleterious processes that may still be occurring, and to capture more advanced pathology or degeneration, we extended the post-stroke time interval to 12 weeks post-surgery in the aged mice. Similar to 8 weeks post-stroke, we saw an abundant amount of Aβ_42_ deposits in the white matter tracts of the 18 mo stroked mice compared to age-matched sham operated mice (Fig. 3e). Quantitation revealed that there was significantly more Aβ_42_ deposition in the ipsilateral hemisphere than in the contralateral hemisphere (Fig. 3f). This finding demonstrates that Aβ_42_ accumulation in response to stroke in aged wt mice is focal, rather than global, and based on its anatomical localization, it appears to localize to the area of axonal degeneration.

It is well established that Aβ_42_ promotes signal transduction mechanisms such as the activation of calpain and stress kinases, and increases p-tau, and that these events can lead to neurite and synapse degeneration, and eventual neuronal death [49, 92, 100]. Ser202 and Thr205, the epitopes on tau that is recognized by the antibody AT8 when they are phosphorylated, are among the tau residues that contribute to the pathological conformation of p-tau [41]. Therefore, we next sought to determine whether Aβ_42_ coincides with the presence of p-tau at these epitopes in the weeks following stroke. As seen in Fig. 3g, the expression of p-tau was significantly higher in the white matter tracts of the 18 mo stroked mice compared to their age-matched sham counterparts, and quantitation revealed that the ipsilateral hemisphere contained significantly more p-tau deposits than the contralateral hemisphere of the stroked mice (Fig. 3h**)**.

Based on morphological assessment, the p-tau staining appeared to be in a linear arrangement consistent with axons. However, to gather more information with regard to the localization of p-tau in our tissue, we performed fluorescent co-staining with GFAP for astrocytes and Iba1 for microglia. We did not detect that p-tau colocalized with the GFAP-labeled astrocytes (Fig. 3i) or with the Iba1-labeled microglia (Fig. 3j).

### Stroke-induced accumulation of Aβ in C57BL/6 mice is associated with expression of BACE1 and NRG1 type III

**Fig. 4:**
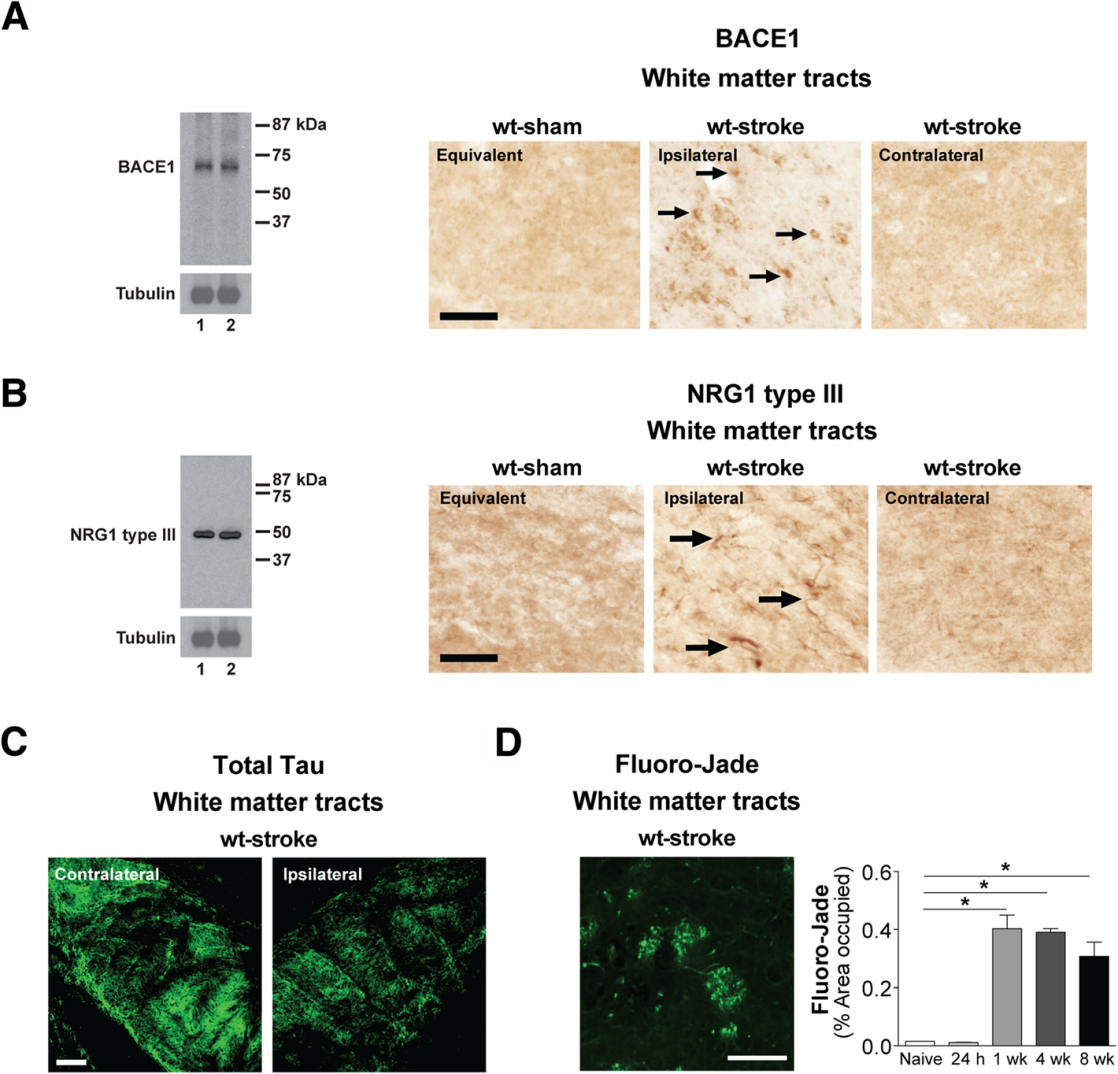
Stroke induces β-secretase (BACE) 1 and neuregulin (NRG) 1 type III expression in the white matter tracts of aged wildtype (wt) mice compared to young adult mice. **a-c** Western blotting with a BACE1 (~70 kDa) or NRG type III (~50 kDa) antibody detected a single band at the appropriate molecular weight in mouse brain lysates for each protein. Representative 20× images (n=3 mice/experimental group) of (**a**) BACE1+ and (**b**) NRG1 type III+ immunostaining (arrows) in the white matter tracts (thalamus-internal capsule) of 18 mo mice at 12 weeks after sham or stroke surgery (Equivalent = area imaged in wt-sham mice that is equivalent to the ipsilateral hemisphere imaged in wt-stroke mice; Contralateral = area imaged in the contralateral hemisphere of wt-stroke mice that is equivalent to the ipsilateral hemisphere of wt-stroke mice). Scale bar, 50 μm (BACE1 and NRG1 type III). Images revealed BACE1 and NRG1 type III expression in the ipsilateral white matter tracts of stroked, but not in sham-operated mice. **c** Representative 10× immunofluorescence images (n=3 mice/experimental group) of total tau+ immunostaining in the ipsilateral and contralateral white matter tracts of wt mice after stroke surgery. Scale bar, 125 μm. **d** Image: Representative 40× Fluoro-Jade staining in the ipsilateral white matter tracts of wt mice after stroke confirms that this is an area of axonal degeneration following DH stroke. Scale bar, 100 μm. Graph: Relative to naïve mice, there is significant Fluoro-Jade staining starting at 1 week (wk) post-stroke, continuing into at least 8 wk post-stroke. Data represent mean ± SEM from n=5 mice/experimental group. *p<0.05

Axonal degeneration and myelin damage distal to stroke injury sites takes months, if not years to resolve [93], and the dysregulation of myelin repair mechanisms has been proposed to contribute to the genesis of AD pathology [11]. A critical myelin homeostasis repair pathway in the CNS involves the upregulation of BACE1 and the cleavage of NRG1 type III [10, 15]. Soluble NRG1 type III β-subunit or transmembrane-bound NRG1 type III then binds to ErbB (epidermal growth factor, EGF) receptors on oligodendrocytes, initiating their myelinating function through the activation of intracellular signaling cascades [19, 39, 87, 89]. However, BACE1 also cleaves AβPP, and causes the release of the pathogenic Aβ peptide [110]. Therefore, because the chronic sequalae of stroke, such as axonal degeneration and chronic inflammation, may place stress on myelin homeostasis, we determined whether BACE1 expression co-localized with the areas of Aβ_42_ deposition following stroke. First, we authenticated the specificity of the BACE1 antibody though Western blotting, and detected a single band at the appropriate molecular weight for mature glycosylated BACE1 of ~70 kDa (Fig. 4a). Then we discovered, as seen in Fig. 4a, that BACE1 was indeed present in the ipsilateral white matter tracts of 18 mo stroked C57BL/6 mice compared to their age-matched sham counterparts at 12 weeks post-surgery. BACE1 was not detected in the contralateral hemisphere of these stroked mice.

To support the hypothesis that BACE1 and NRG1 type III are part of a chronically activated myelin repair mechanism following stroke, we then tested if NRG1 type III expression is chronically increased in stroked wt mice, and if it colocalizes with areas of BACE1 expression and Aβ_42_ deposition. First, we authenticated the specificity of the NRG1 type III antibody though Western blotting, and detected a single band at the appropriate molecular weight of ~50 kDa (Fig. 4b). We then found, as depicted in Fig. 4b, that NRG1 type III was present in the ipsilateral white matter tracts of the 18 mo stroked C57BL/6 mice compared to their age-matched sham counterparts at 12 weeks post-surgery. NRG1 type III was not detected in the contralateral hemisphere of these stroked mice. Taken together, these findings lend support to the possibility that deposition of Aβ_42_ following stroke is a by-product of a chronically activated myelin repair process in areas of axonal degeneration in aged C57BL/6 mice.

To confirm that the expression of BACE1 and NRG1 type III in the white matter tracts of the wt stroked mice was correlating with the area of axonal degeneration, we performed immunostaining for total tau and Fluoro-Jade staining. The total tau immunostaining revealed a chronic loss of total tau immunoreactivity in the ipsilateral white matter tracts compared to the contralateral white matter tracts of the aged wt stroked mice (Fig. 4c), and the Fluoro-Jade staining revealed a delayed and sustained area of degenerating axons in the ipsilateral white matter tracts (Fig. 4d).

### Stroke exacerbates motor, cognitive, and psychological behavioral deficits in aged hAPP-SL mice

**Fig. 5:**
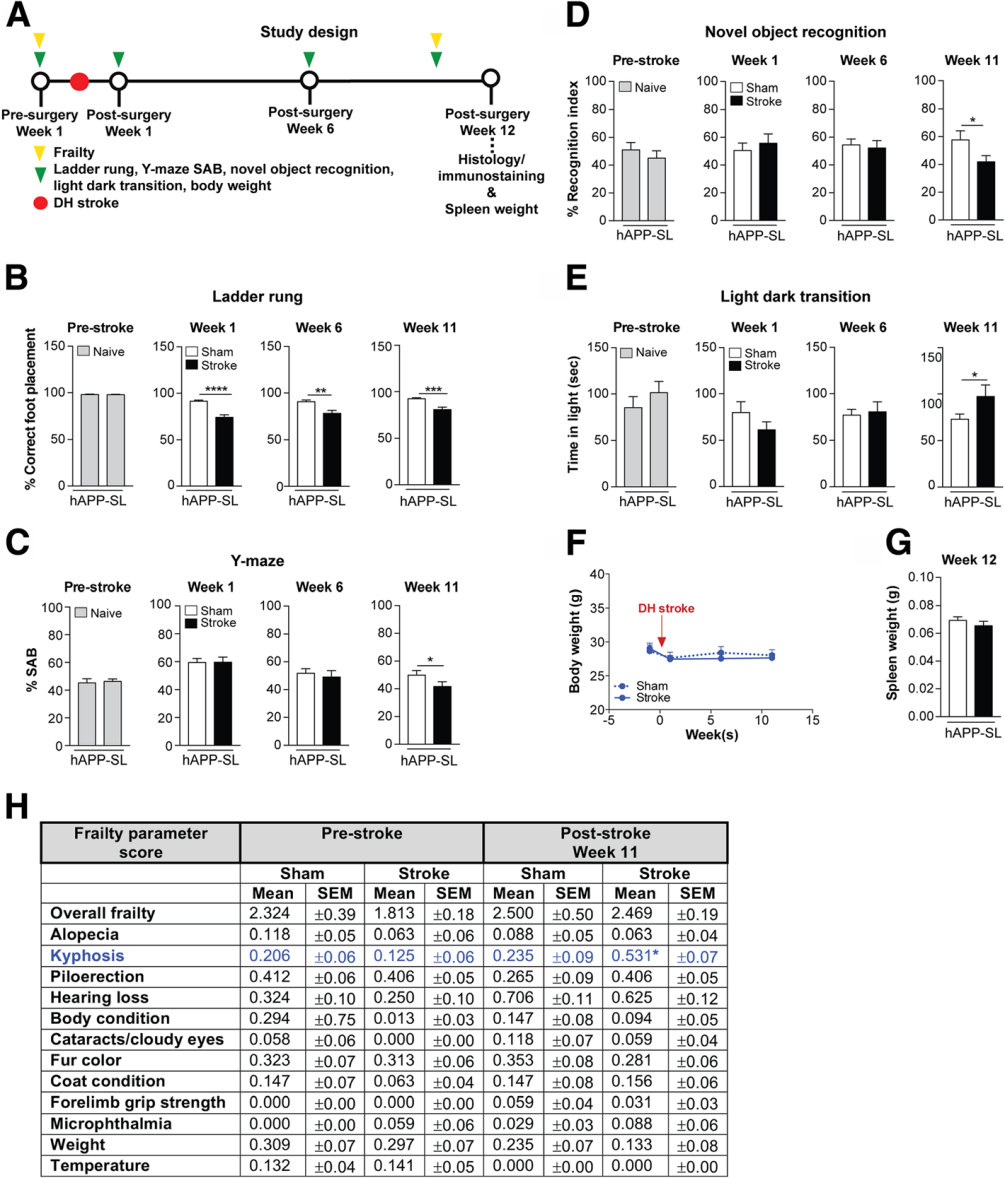
Stroke exacerbates behavioral deficits in aged hAPP-SL mice on tests of motor, cognition, and anxiety. **a** Study design: 18 month-old (mo) hAPP-SL mice were assessed on the ladder rung, Y-maze spontaneous alternation behavior (SAB), novel object recognition, and light dark transition tests prior to a distal hypoxic (DH) stroke or sham surgery at the indicated timepoints. Mice were also weighed at each indicated timepoint. Mice were euthanized and brains were harvested for histology and immunostaining at 12 weeks post-surgery, along with spleen for organ weight. **b** Motor ability on the ladder rung test was assessed at 1-week pre-surgery, and at 1 week, 6 weeks, and 11 weeks post-surgery. At 1 week prior to a stroke, there was no difference in motor performance in the naïve hAPP-SL mice, as mice displayed a similar number of acceptable (<12% error) baseline foot placements on the rungs. These naïve mice would later be assigned into the sham or stroke experimental groups. At 1 week post-surgery, stroked hAPP-SL mice exhibited an enhanced motor deficit, as they displayed a significantly fewer number of correct foot placements relative to sham-operated hAPP-SL mice. Significant motor deficits continued to manifest in stroked hAPP-SL mice at 6 and 11 weeks post-surgery compared to sham-operated hAPP-SL mice at those timepoints. **c** Cognitive function using the Y-maze SAB test was assessed at 1 week pre-stroke, and at 1 week, 6 weeks, and 11 weeks post-surgery. At 1 week prior to surgery, there was no difference in cognitive function in naïve hAPP-SL mice, as mice displayed similar levels of spontaneous alternations. At 1 and 6 weeks post-surgery, there was no difference in the cognitive status of sham- and stroke-operated hAPP-SL mice. However, at 11 weeks post-surgery, stroked hAPP-SL mice exhibited aggrevated short-term spatial memory impairment, as they displayed significantly less spontaneous alternations compared to sham-operated hAPP-SL mice. **d** Cognitive function using the novel object recognition test was assessed at 1 week pre-stroke, and at 1 week, 6 weeks, and 11 weeks post-surgery. At 1 week prior to surgery, there was no difference in cognitive function in naïve hAPP-SL mice, as mice displayed similar recognition indexes, which corresponds to similar exploration time for an unfamiliar (novel) and a familiar object. At 1 and 6 weeks post-surgery, there was no difference in the cognitive status of the sham- and stroke-operated hAPP-SL mice. However, at 11 weeks post-surgery, the stroked hAPP-SL mice exhibited worsened intermediate recognition memory impairment, as they displayed significantly lower recognition indexes calculated from less time spent distinguishing and exploring an unfamiliar/novel object compared to the sham-operated hAPP-SL mice. **e** Using the light dark transition test, we assessed mice on the anxiety-impulsivity spectrum of behavior at 1 week pre-surgery, and at 1 week, 6 weeks, and 11 weeks post-surgery. At 1 week prior to surgery, there was no difference in the amount of time spent in the light, open (intimidating space) versus the dark, enclosed (safe space) arenas of the chamber in the naïve hAPP-SL mice. At 1 week prior to surgery, the amount of time spent in each arena remained similar between sham- and stroke-operated hAPP-SL mice, and this pattern was seen at 6 weeks post-surgery. However, at 11 weeks post-surgery, stroked hAPP-SL mice spent significantly more time in the light arena than sham-operated hAPP-SL mice, suggesting that stroke initiated behavioral impulsivity or a lack of inhibition in the hAPP-SL mice by reducing their anxiety of open spaces. **f** No significant weight changes were seen in the sham- and stroke-operated experimental hAPP-SL mouse groups at pre- and post-surgery timepoints. **g** There was no spleen weight difference between experimental groups at 12 weeks post-surgery. **h** There was no significant difference in any of the selected frailty outcomes depicted, with the exception of kyphosis, in the 18 mo sham- versus stroke-operated hAPP-SL mice at pre- and post-surgery timepoints. Data represent mean ± SEM. *p<0.05, **p<0.01, ***p<0.001, and ****p<0.0001

In the first part of this study, we used aged C57BL/6 mice as one model of post-stroke mixed dementia. To complement this model, in the second part of the study, we used a combination of age, stroke, and Tg AβPP mice, which are an *in vivo* chronic Aβ deposition and degeneration model of AD (see Study design in Fig. 5a). Evidence in humans suggest that vascular risk factors such as stroke increase the risk of developing AD [91], with a synergistic effect in some cases [32]. Furthermore, in human mixed dementia, most patients have more varied pathology with respect to Aβ accumulation, brain atrophy, and neurodegeneration than typical “pure” AD patients [43]. Therefore, this second model of post-stroke mixed dementia is useful for determining how a typical AD phenotype evolves into an atypical subtype over time, or vice versa, when interacting with the chronic sequelae of stroke.

First, we assessed the motor ability of aged hAPP-SL mice, using the ladder rung test following an ischemic stroke with a chronic post-surgery timeframe of 12 weeks. Baseline data showed no impairment in ladder performance before surgery in the 18 mo hAPP-SL mice (Fig. 5b). The naïve mice were then allocated into experimental groups and given either a stroke or sham surgery. At 1-week post-surgery, there was a significant difference in motor function between experimental groups, as the stroked hAPP-SL mice performed significantly worse compared to their age-matched sham counterparts. This difference in motor function was still present when mice were tested at 6 weeks post-surgery and at 11 weeks post-surgery.

We have previously demonstrated that AβPP mice exhibit a host of behavioral deficits (contextual fear conditioning, delayed-matching-to-place in a modified Barnes maze and Morris water maze, Y-maze SAB, T-maze, NOR, and social novelty compared to their age-matched wt littermates on a C57BL/6 background [25, 33, 48, 64, 74, 76, 77, 79]). Therefore, we proceeded to examine the effect of stroke on the cognitive status of aged hAPP-SL mice by using the Y-maze SAB to assess spatial working memory. As seen in Fig. 5c, spontaneous alternation of Y-maze arm choices between naïve hAPP-SL mice, which were later allocated into stroke or sham experimental groups, were indistinguishable from each other pre-surgery, and continued to be indistinguishable during weeks 1-6 post-surgery. However, at week 11 post-surgery, significantly poorer spontaneous alternation performance was detected in the stroke- compared to the sham-operated hAPP-SL mice.

To corroborate these findings, NOR testing was also performed. In NOR testing, mice tend to spend more time exploring a novel object relative to a familiar object. Because the inter-trial-interval (ITI) here was 4 hours, this ITI timeframe has been reported to test intermediate memory retention [9] as opposed to spatial working memory from the SAB test. Also, the NOR test is a test of recognition memory. Naïve hAPP-SL mice, which were later allocated into stroke or sham experimental groups, had similar recognition indexes (RI) to each object at the pre-surgery timepoint (Fig. 5d). Recognition indexes between stroke- and sham-operated mice were also equivalent during week 1-6 post-surgery. However, a loss of object recognition ability became apparent in the stroked hAPP-SL mice at 11 weeks post-surgery, as they had a significantly lower RI compared to the sham-operated hAPP-SL mice.

Psychological disturbances, including depression, anxiety-impulsivity, aggression-submission, and psychosis are increasingly recognized as a manifestation of the pathological features present in AD and other age-related neurodegenerative diseases [12, 45, 71, 95]. Adult hAPP-SL mice display significant psychological disturbances such as anxiety- and submissive-like behaviors, and sensorimotor gating deficits compared to their wt littermates (Nguyen TV et al., unpublished data). Here, we determined the chronic impact of stroke on a psychological measurement of anxiety-impulsivity in aged hAPP-SL mice. The LDT test measures the spontaneous exploratory behavior of mice in response to mild stressors, such as a novel environment and brighter lighting after some habituation [50]. As seen in Fig. 5e, naïve hAPP-SL mice, which were later allocated into stroke or sham experimental groups, experienced comparable levels of exploration in the light chamber pre-surgery. The amount of exploration time in the light chamber was also comparable between the stroke- and sham-operated hAPP-SL mice during weeks 1-6 post-surgery. However, at 11 weeks post-surgery, the stroked hAPP-SL mice spent significantly more time in the light chamber compared to the sham-operated hAPP-SL mice. This increased time spent in the open, light, and what should be an intimidating arena, by the stroked hAPP-SL mice, supports a display of behaviors resembling the impulsive, uninhibited, or risk-taking behaviors exhibited by patients with dementia, including frontotemporal dementia and AD [54].

Because of reports suggesting changes in body and spleen weights (correlated with inflammatory signals), and the development of frailty in age-related dementias, such as AD [35, 78, 81, 96, 106], we evaluated the impact of stroke on these physiological parameters in aged hAPP-SL mice. However, we did not detect a significant difference in the body weights of both the stroke- and sham-operated hAPP-SL mice at pre- and post-surgery timepoints (Fig. 5f). Likewise, there was no significant difference in the spleen weights of both the stroke- and sham-operated hAPP-SL mice at 12 weeks post-surgery (Fig. 5g). Furthermore, there was no significant difference in any of the selected frailty outcomes indicated, with the exception of kyphosis, in the stroke- versus sham-operated hAPP-SL mice at pre- and post-surgery timepoints (Fig. 5h). Overall, the hAPP-SL mice appeared physiologically similar at the end of the study regardless of having undergone a stroke or sham procedure.

### Stroke exacerbates global cholinergic degeneration in aged hAPP-SL mice

**Fig. 6:**
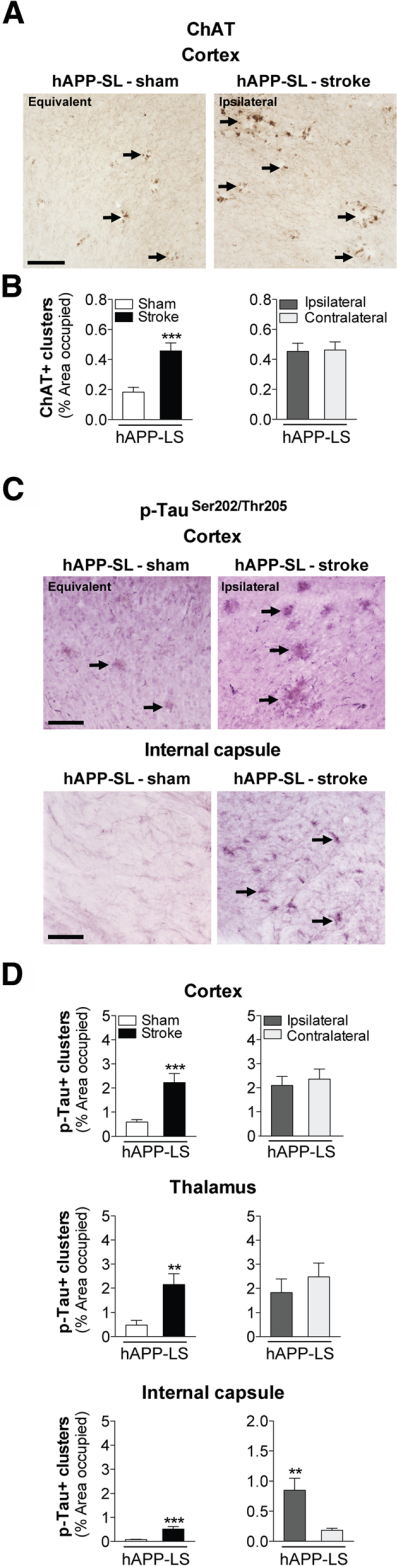
Stroke increases cholinergic neurodegeneration and levels of tau phosphorylation (p) in aged hAPP-SL mice. **a** Representative 10× images of choline acetyltransferase (ChAT)-immunolabeled dystrophic neurites (arrows) in the primary somatosensory cortex of 18 mo sham- or stroke-operated hAPP-SL mice (Equivalent = area imaged in wt-sham mice that is equivalent to the ipsilateral hemisphere imaged in wt-stroke mice). Scale bar, 125 μm. **b** Quantification revealed that relative to sham-operated hAPP-SL mice, the area occupied by cholinergic dystrophic neurites in the cortex was significantly higher in the stroked hAPP-SL mice; no significant difference in the amount of cholinergic dystrophic neurites was found between the ipsilateral versus contralateral cortex. **c** Representative 10× images of AT8 (p-tau^Ser202/Thr205^)-immunolabeled dystrophic neurites (arrows) in the primary somatosensory cortex of 18 mo sham- or stroke-operated hAPP-SL mice (Equivalent = area imaged in wt-sham mice that is equivalent to the ipsilateral hemisphere imaged in wt-stroke mice). Scale bar, 125 μm. **d** Quantification revealed that relative to sham-operated hAPP-SL mice, the area occupied by p-tau+ dystrophic neurites was significantly higher in the cortex (top graph), thalamus (middle graph), and internal capsule (bottom graph) of the stroked hAPP-SL mice; no significant difference in the amount of p-tau+ dystrophic neurites was found between the ipsilateral versus contralateral cortex and thalamus, although the ipsilateral internal capsule showed significantly more p-tau+ dystrophic neurites than the contralateral region. **p<0.01 and ***p<0.001

Given that chronic stroke exacerbates behavioral deficits in aged hAPP-SL mice, we evaluated whether these manifestations are correlated with neurodegeneration. Adult hAPP-SL mice exhibit cholinergic neurite degeneration in the basal forebrain, as well as a loss of projection fibers and prominent dystrophic neurites in the cortex [48, 64, 85]. Accumulation of Aβ in AD is associated with the appearance of dystrophic neurites [57], which likely impair neuronal and synaptic function. Dystrophic neurites are swollen, bulbous, and tortuous processes that cluster together [13], are intrinsic to degenerative changes that result from cytoskeletal derangement [36], manifest early in AD [14], and precede neuronal death [31]. Here, we assessed whether stroke impacts cholinergic dystrophic neurites in aged hAPP-SL mice. As seen in Fig. 6a, clusters of cholinergic dystrophic neurites appeared more abundant, as indicated by significantly more ChAT-labeled dystrophic neurites occupied in the cortex of the 18 mo stroked mice compared to the sham-operated hAPP-SL mice (Fig. 6b). There was no difference in the percentage occupied by dystrophic neurite clusters in the ipsilateral versus the contralateral hemisphere of the stroked hAPP-SL mice, suggesting not only an exacerbated effect of a stroke on cholinergic pathology in hAPP-SL mice, but also a global effect.

### Stroke exacerbates global tau pathology in aged hAPP-SL mice

Previous studies report that cholinergic dystrophic neurites are found in the vicinity of tau and amyloid deposits in the cortex of adult hAPP-SL mice [48, 64]. Dystrophic neurites have been shown to contain abnormal filaments of hyperphosphorylated tau proteins [8, 97]. Here, we assessed whether stroke impacts p-tau+ dystrophic neurites in aged hAPP-SL mice up to 12 weeks post-stroke. As seen in Fig. 6c, clusters of p-tau+ containing dystrophic neurites were more abundant in the 18 mo stroked hAPP-SL mice compared to the sham-operated hAPP-SL mice, as indicated by significantly more p-tau+ dystrophic neurites in the cortex and white matter tracts of the internal capsule of these mice (Fig. 6d). There was also significantly more p-tau+ staining in both the white matter tracts of the thalamus and the internal capsule of the stroked hAPP-SL mice versus the sham-operated hAPP-SL mice. Furthermore, for each brain region, there was no difference in the area occupied by p-tau+ dystrophic neurite clusters in the ipsilateral compared to the contralateral hemisphere of the stroked hAPP-SL mice, suggesting once more not only an exacerbated effect of a stroke on AD-related pathology in hAPP-SL mice, but also a global effect.

### Stroke exacerbates global Aβ burden in aged hAPP-SL mice

**Fig. 7:**
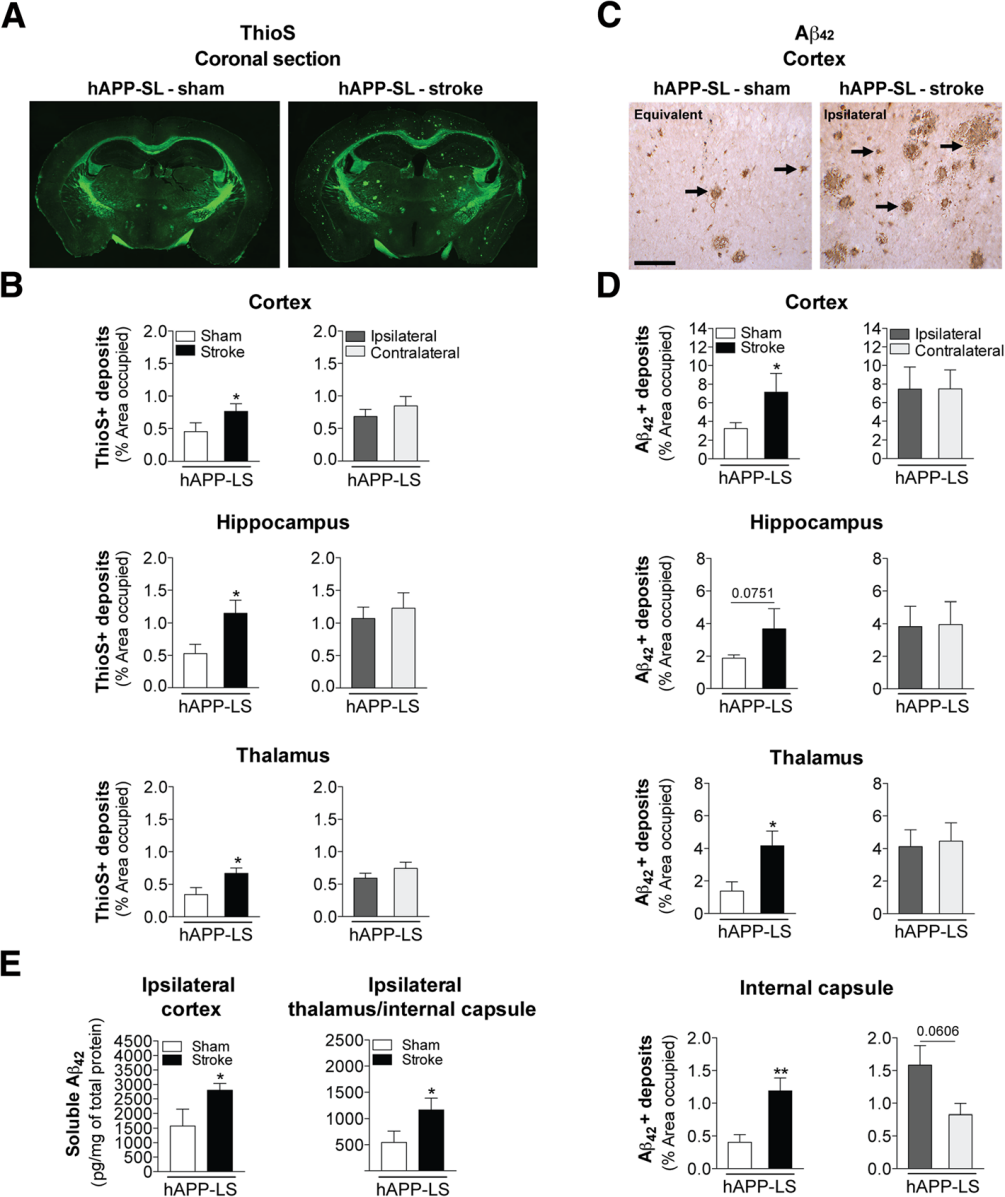
Stroke exacerbates amyloid plaque burden and soluble Aβ_42_ levels in aged stroke hAPP-SL mice. **a** Representative 4× stitched images of Thioflavin S (ThioS)-stained coronal brain sections of 18 mo sham- or stroke-operated hAPP-SL mice. **b** Quantification revealed that relative to sham-operated hAPP-SL mice, the area occupied by ThioS-labeled amyloid plaques was significantly greater in the cortex (top graph), hippocampus (middle graph), and thalamus (bottom graph) of the stroked hAPP-SL mice; no significant difference in the amount of amyloid plaques was found between the ipsilateral versus contralateral hemisphere. **c** Representative 10× images of Aβ_42_-immunolabeled deposits (arrows) in the primary somatosensory cortex of the 18 mo sham- or stroke-operated hAPP-SL mice (Equivalent = area imaged in wt-sham mice that is equivalent to the ipsilateral hemisphere imaged in wt-stroke mice). Scale bar, 125 μm. **d** Quantification revealed that relative to sham-operated hAPP-SL mice, the area occupied by Aβ_42_+ deposits was significantly higher in the cortex (top graph), thalamus (bottom middle graph), and internal capsule (bottom graph) of the stroked hAPP-SL mice (p value for the hippocampus shown in the top middle graph was 0.0751); no significant difference in the amount of Aβ_42_+ deposits was found between the ipsilateral versus contralateral hemisphere. **e** A single-plex immunoassay of tissue samples from the ipsilateral cortex or thalamus/internal capsule regions showed that in hAPP-SL mice, significantly higher levels of soluble Aβ_42_ are found in stroked mice compared to sham-operated mice. Data represent mean ± SEM. *p<0.05 and **p<0.01

Previous work has shown that increased p-tau expression in hAPP-SL mice results from Aβ-induced signal transduction mechanisms, including activation of calpain and stress kinases (cdk5, GSK3β, c-Jun, and p38) [64, 107]. Because there was significantly more p-tau expression in the stroke- versus sham-operated hAPP-SL mice, we wanted to determine whether abnormally high p-tau expression correlated with stroke-induced exacerbation of Aβ pathology at 12 weeks post-surgery. Therefore, we examined amyloid plaque distribution in ThioS-stained brain sections from 18 mo hAPP-SL mice given stroke or sham surgery. ThioS primarily stains fibrillary forms of Aβ, and notably, ThioS pathology was not detected in the young adult or aged wt C57BL/6 mice that underwent stroke surgery. However, as seen in Fig. 7a, we found more abundant amyloid plaques, as detected by ThioS’s intrinsic fluorescence, in the brains of the stroked hAPP-SL mice compared to the sham-operated hAPP-SL mice. Quantitation revealed a significantly higher percentage of cortical and hippocampal areas occupied by ThioS-stained deposits in the stroke- versus sham-operated hAPP-SL mice (Fig. 7b). Also, there were significantly more ThioS-stained deposits in both the white matter tracts of the thalamus of the stroked versus sham hAPP-SL mice. For each brain region, there was no significant difference in the area occupied by ThioS-stained deposits in the ipsilateral versus the contralateral hemisphere of the stroked hAPP-SL mice. This again suggests not only an exacerbated effect of stroke on amyloid pathology, as revealed by ThioS staining, in hAPP-SL mice, but also a global effect.

Though ThioS stains primarily fibrillary forms of Aβ, it may also stain some dystrophic neurites [61]. Therefore, to more accurately determine the effect of stroke on Aβ pathology, we next probed for the pathological Aβ_1-42_ peptide using a DAB-linked secondary antibody to a specific Aβ_42_ antibody. We detected abundant Aβ_42_-immunolabeled deposits in the cortex of the 18 mo stroke- compared to sham-operated hAPP-SL mice at 12 weeks post-surgery (Fig. 7c). Quantitation showed significantly more Aβ_42_+ deposits in the cortex, thalamus, and internal capsule (p value of 0.0751 in the hippocampus) of the stroke- versus sham-operated hAPP-SL mice (Fig. 7d). There was no significant difference in the area occupied by Aβ_42_+ deposits in the ipsilateral versus the contralateral hemisphere of the stroked hAPP-SL mice in the cortex, hippocampus, or thalamus, but there was a non-significant trend (*p* value of 0.0606) of an increase in the internal capsule. These data again suggest not only an exacerbated effect of stroke on Aβ_42_ pathology in hAPP-SL mice, but also a global effect.

To more precisely measure the concentration of Aβ_42_ in hAPP-SL mice after stroke and sham surgery, we quantified Aβ_42_ in dissected ipsilateral brain region homogenates 12 weeks after surgery. Using a single-plex immunoassay kit and the Luminex technology platform, we found significantly higher levels of soluble Aβ_42_ in the ipsilateral cortex and the ipsilateral white matter (which included the thalamus and internal capsule) of the stroke- compared to sham-operated hAPP-SL mice (Fig. 7e). Taken together, the data so far suggest that stroke chronically exacerbates neurodegeneration in hAPP-SL mice, possibly through enhanced p-tau and Aβ_42_ pathology.

### BACE1 expression is chronically increased in aged hAPP-SL mice following stroke

**Fig. 8:**
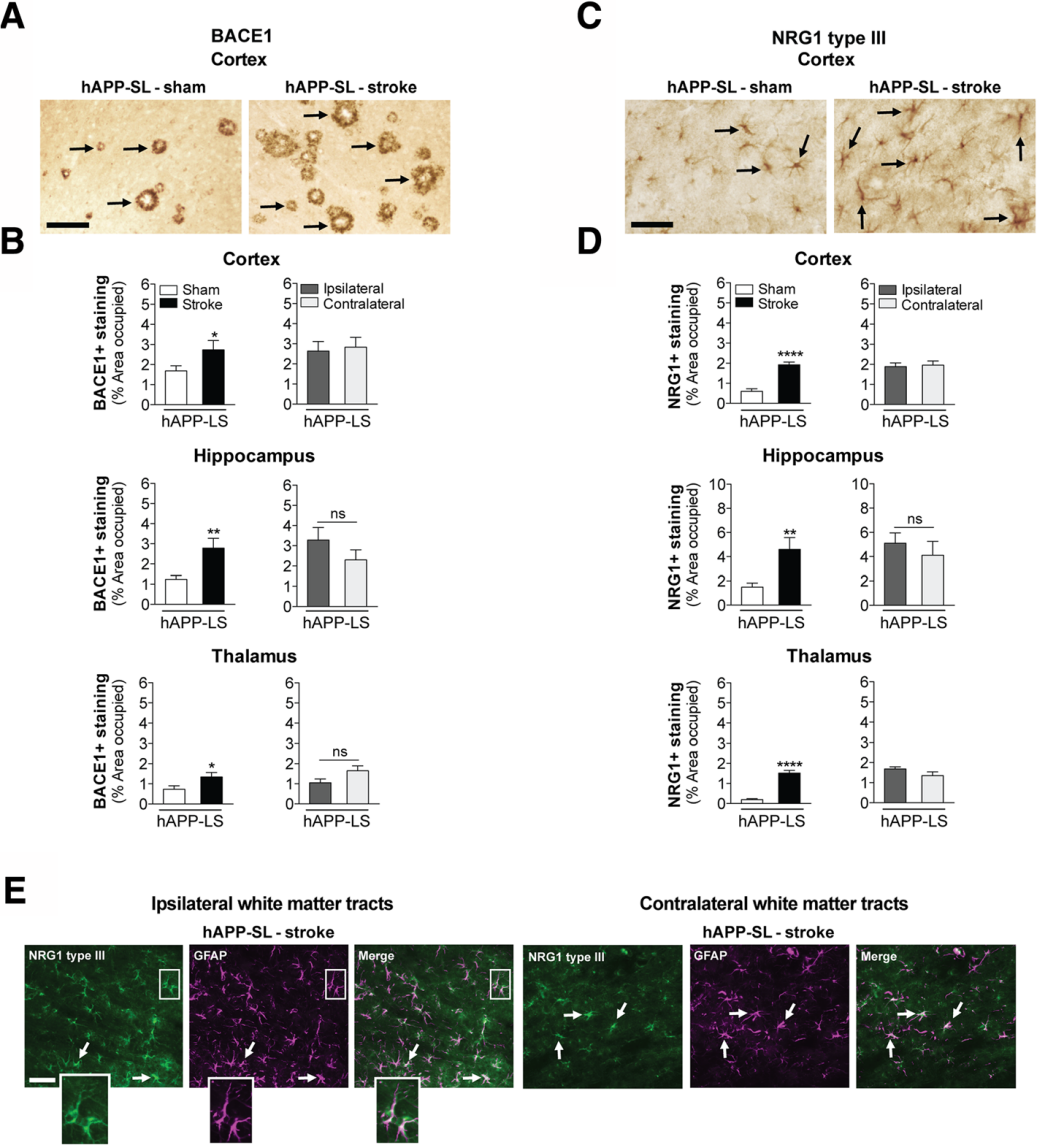
Stroke increases β-secretase (BACE) 1 and neuregulin (NRG) 1 type III immunostaining in aged hAPP-SL mice. **a** Representative 10× images of BACE1+ immunostaining (arrows) in the primary somatosensory cortex of 18 mo sham- or stroke-operated hAPP-SL mice. Scale bar, 125 μm. **b** Quantification revealed that relative to sham-operated hAPP-SL mice, the area occupied by BACE1+ staining was significantly higher in the cortex (top graph), hippocampus (middle graph), and thalamus (bottom graph) of the stroked hAPP-SL mice; no significant difference in the amount of BACE1+ staining was found between the ipsilateral versus contralateral hemisphere. **c** Representative 20× images of NRG1 type III+ immunostaining (arrows) in the primary somatosensory cortex of 18 mo sham- or stroke-operated hAPP-SL mice. Scale bar, 125 μm. **d** Quantification revealed that relative to sham-operated hAPP-SL mice, the area occupied by NRG1 type III+ staining was significantly higher in the cortex (top graph), hippocampus (middle graph), and thalamus (bottom graph) of the stroked hAPP-SL mice; no significant difference in the amount of NRG1 type III+ staining was found between the ipsilateral versus contralateral hemisphere. Data represent mean ± SEM. *p<0.05, **p<0.01, and ****p<0.0001. **e** Representative 40× fluorescence images (n=3 mice/experimental group) from the 18 mo stroked hAPP-SL mouse sections showing NRG1 type III staining (green) within cells staining for the astrocytic marker, glial fibrillary acidic protein (GFAP, magenta), in the ipsilateral and contralateral white matter tracts (thalamus-internal capsule). Magnified outsets show GFAP+ astrocytes colocalized with NRG1 type III. Scale bar, 50 μm

In aged wt mice, we detected significant BACE1 accumulation in the white matter tracts of the ipsilateral hemisphere of the stroked compared to the sham-operated C57BL/6 mice (Fig. 4a). Our findings parallel those of Hilunen and colleagues who reported a similar result after focal cerebral ischemia in adult rats [37]. Here, we determined whether a chronic sequela of stroke in hAPP-SL mice is a global increase in BACE1, thereby resulting in a global increase in production of Aβ_42_. As seen in Fig. 8a, there was more BACE1 accumulation in the cortex of the 18 mo stroked-hAPP-SL mice compared to the sham-operated hAPP-SL mice at 12 weeks post-surgery. Quantitation showed significantly more BACE1+ deposits in the cortex, hippocampus, and thalamus of stroked versus sham-operated hAPP-SL mice (Fig. 8b). There was no significant difference, however, in the percentage area occupied by BACE1 staining in the ipsilateral versus the contralateral hemisphere of the stroked hAPP-SL mice, indicating that there is indeed a global increase in BACE1 expression in hAPP-SL mice that lasts for weeks following stroke.

### NRG1 type III expression is chronically increased in aged hAPP-SL mice following stroke

As previously discussed, soluble or transmembrane-bound NRG1 type III binds to ErbB receptors on oligodendrocytes as part of an endogenous myelin homeostasis mechanism. This induces oligodendrocyte myelination through receptor-mediated signaling, and it requires the cleavage of NRG1 type III by BACE1. Increased levels of NRG1 have been detected in Schwann cells of the PNS in response to spinal cord injury [2, 3], however, the impact of chronic stroke on NRG1 type III expression was previously unknown. Data in Fig. 4b show that NRG1 type III expression is present in the white matter tracts of the ipsilateral hemisphere of aged wt mice at 12 weeks post-stroke, and here, we determined the chronic impact of stroke on NRG1 type III expression in aged hAPP-SL mice. As seen in Fig. 8c, expression of NRG1 type III was more abundant in the cortex of the 18 mo stroked hAPP-SL mice compared to the sham-operated hAPP-SL mice at 12 weeks post-surgery. Quantitation showed significantly more NRG1 type III expression in the cortex, hippocampus, and thalamus of the stroked versus sham hAPP-SL mice (Fig. 8d). Similar to BACE1 expression, there was no significant difference in the percent area of each brain region occupied by NRG1 type III staining in the ipsilateral versus the contralateral hemisphere of the stroked hAPP-SL mice, indicating that there is a global rather than a focal increase in NRG1 type III expression in hAPP-SL mice.

NRG1 type III, either in its soluble or transmembrane-bound form, has been reported to be produced by astrocytes, [20, 29, 86, 88, 90]. Therefore, we next performed NRG1 type III and astrocyte (GFAP) co-staining. Assessment of GFAP-immunolabeled astrocytes in the white matter tracts (thalamus/internal capsule) from tissue samples of the 18 mo stroked hAPP-SL mice revealed medium to high levels of co-staining of NRG1 type III with GFAP in both the ipsilateral and contralateral hemispheres (Fig. 8e).

## Discussion

In this study, we provide evidence that AD-related pathology is a chronic sequela of ischemic stroke in two mouse models of post-stroke mixed dementia. To our knowledge, this is one of the most comprehensive studies investigating the long-term impact of ischemia on the development of the pathology related to AD to date, and the first study to compare differences in stroke-induced pathology in wt and hAPP-SL mice. Importantly, our post-stroke mixed dementia models are able to track the development of pathology related to AD over the period of weeks and months following stroke in mice. This is because the stroke model employed has low mortality and variability, even though it causes a sizeable infarct that encompasses approximately 25% of the ipsilateral hemisphere.

In wt mice, we found that ischemic stroke impairs motor recovery and accelerates the onset of cognitive impairment in aged compared to young adult C57BL/6 mice. This finding correlated with more extensive brain atrophy, cholinergic neurodegeneration, and deposition of p-tau and Aβ_42_ in the ipsilateral white matter tracts of the 18 mo stroked C57BL/6 mice compared to their 3 mo counterparts. Furthermore, increased levels of p-tau and Aβ_42_ were still present at 12 weeks post-stroke in a separate cohort of 18 mo C57BL/6 mice that underwent stroke surgery. ThioS pathology, which labels fibrillary forms of Aβ, was not detected in the adult or aged wt C57BL/6 mice that underwent stroke surgery. These findings demonstrate that in aged animals, stroke by itself can cause a subset of the brain abnormalities associated with AD.

**Fig. 9:**
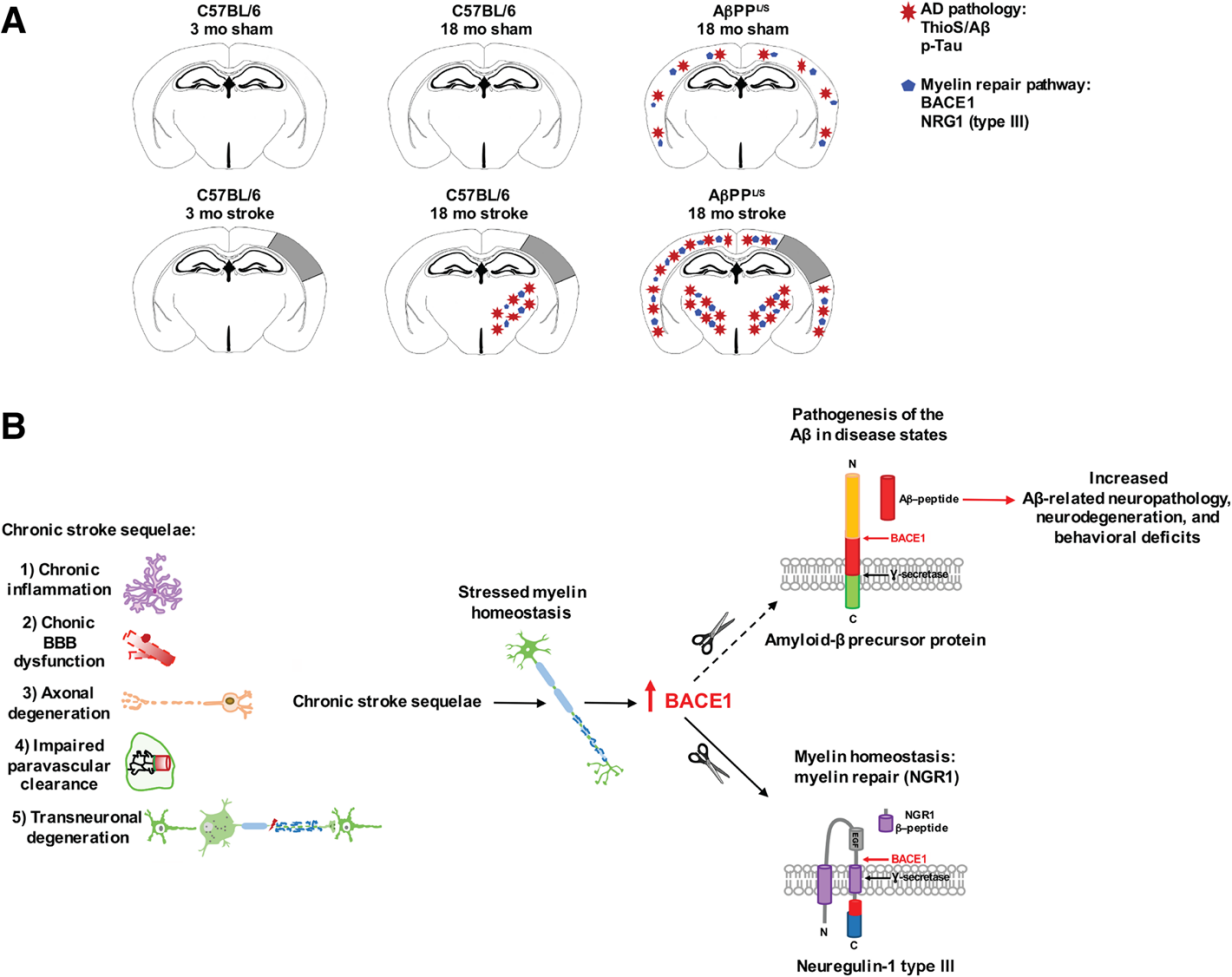
Schematic representations summarizing key findings of our study. **a** In this study, we provide evidence that there is overlap of pathology (amyloid plaques, and Aβ_42_ and p-tau deposition) associated with AD following stroke with key components (BACE1 and NRG1 type III) of a myelin repair pathway in aged stroked wt and stroked hAPP-SL mice. **b** Working model proposing AD-associated pathology as a chronic sequela of ischemic stroke: chronic inflammation, chronic BBB dysfunction, axonal degeneration, impaired paravascular clearance, and transneuronal degeneration are all chronic sequalae of stroke that may result in stressed myelin homeostasis. BACE1’s role as a protease that cleaves NRG1 type III at its β-site is critical in the formation of myelin sheaths by oligodendrocytes, and is a key regulator of myelination of axons in the central nervous system [10]. BACE1’s cleavage of NRG1 type III initiates γ-secretase cleavage of NRG1 type III at its γ-site. However, in addition to NRG1 type III, BACE1 has high cleavage affinity for the Aβ precursor protein (AβPP). Therefore, the chronic sequelae of stroke may be causing myelin repair mechanisms involving NRG1 type III and BACE1 to be racheted up, resulting in the inadvertent cleavage of AβPP by BACE1 and γ-secretase, and the abnormal generation of amyloidogenic Aβ peptides

In the hAPP-SL mice, however, we found that stroke worsened a broader array of the stereotypical hallmarks of AD-like pathology, and that worsened pathology was evident throughout both hemispheres of the brain. In both of these models, the stroke-induced amyloid and tau deposition co-localized with AβPP cleavage enzyme, BACE1, and myelin-associated protein, NRG1 type III (Fig. 9a), both of which are critical for myelin repair. Based on these findings, we propose that a myelin homeostasis mechanism is focally malfunctioning or overwhelmed in response to stroke in aged wt mice, and is globally malfunctioning or overwhelmed in hAPP-SL mice. Due to a consequence of advanced age or having the hAPP-SL transgene, this is leading to the inadvertent cleavage of AβPP, Aβ deposition, and subsequent exacerbated neurodegeneration.

This possibility is in agreement with the hypothesis put forth by Bartzokis and colleagues that AD pathology may be the result of a defective homeostatic response to age-related myelin breakdown [11, 55]. This hypothesis suggests that because in addition to NRG1 type III, BACE1 has high cleavage affinity for AβPP, over activation of the NRG1/BACE1 myelin repair mechanism may result in the inadvertent cleavage of AβPP in aged animals (Fig. 9b). It is not fully understood which cell type or types produce NRG1 type III following stroke, however, recent data, as well as the data presented here, support reactive astrocytes as one source [20, 29, 86, 88, 90].

An important observation from our findings is that there was a difference in the development of AD-like pathology following stroke in the 3 mo and 18 mo wt mice. Unlike their aged counterparts, the 3 mo stroked C57BL/6 mice did not exhibit Aβ or p-tau pathology following stroke. Why the chronic consequences of stroke impacted AD-related pathology differently in the young adult versus the aged C57BL/6 mice could be due to myelin repair processes becoming less efficient or malfunctioning with increased age [62]. Alternatively, with advanced age comes an attenuated inflammatory response following stroke [84]. As a consequence, there may be diminished clearance of myelin debris in the repair process in the area of axonal degeneration by microglia/macrophages in the 18 mo stroked mice, thereby leading to a hyperactive or prolonged myelin repair process.

Another important observation from our findings is the difference in the regional distribution of Aβ and p-tau in the brains of the aged wt mice and the aged hAPP-SL mice. In the wt mice, AD-like pathology was primarily confined to the ipsilateral white matter tract areas, mainly including the thalamus and internal capsule, whereas in the hAPP-SL mice there was exacerbated distribution in grey and white matter areas of both hemispheres. We propose that this could be due to myelin repair being less effective in aged C57BL/6 mice than in younger mice, but not severely so. Therefore, perhaps in aged wt mice, AD-associated pathological amyloid and tau species only develop in an area of maximal myelin repair, such as the area of axonal degeneration. On the other hand, myelin homeostasis may be closer to a tipping point that leads to the deposition of Aβ throughout the brain in aged hAPP-SL mice. Following a stroke, the combined impact of multiple chronic sequelae of stroke may send myelin homeostasis over this tipping point in more brain regions in hAPP-SL mice.

Examples of chronic consequences of stroke, separate from axonal degeneration, that could be leading to the more widespread overload of this tipping point in hAPP-SL mice, include chronic inflammation, prolonged blood brain barrier (BBB) impairment, and impaired paravascular clearance. For instance, recently published data from our research group demonstrate that CNS infarcts in both humans and mice are sites of sustained expression of proinflammatory cytokines/chemokines, sustained foamy macrophage responses, and the sustained presence of T- and B-lymphocytes for at least 7 weeks following stroke [23, 63]. These responses are imperfectly sequestered from the rest of the brain by glial scars [108]. Another chronic consequence of stroke is prolonged BBB impairment, for instance our previously published data from adult C57BL/6 mice demonstrate that neovascularization following stroke results in the formation of a network of new blood vessels within the infarct that do not mature into vessels with full BBB competency [108]. Finally, studies by Wang and colleagues using micro-infarcts, recently demonstrated that paravascular clearance is perturbed in the vicinity of the infarct following stroke [98]. A consequence of this impairment may be the accumulation of potentially toxic molecules that could promote protein aggregation and inflammation. These “trapped” molecules may be proinflammatory cytokines and cytotoxic extracellular Aβ, or extracellular tau released into the brain interstitial fluid as a result of axonal damage [40] [56, 60]). Needless to say, each of these processes could also be contributing to AD pathogenesis following stroke via a mechanism independent of their impact on myelin homeostasis. However, they may have a pervasive impact on myelin homeostasis if they are sending toxic factors widespread and indiscriminately throughout the brain.

As a final caveat, a potential limitation of the models of post-stroke mixed dementia presented here is that the accumulation of amyloid and tau in the white matter is not a typical characteristic of AD, either with or without vascular brain injury. However, using sensitive imaging techniques several independent groups have recently reported elevated Aβ burden in the white matter of AD patients independent of cortical plaque severity [16], as well as in preclinical AD [72].

## Conclusions

In conclusion, despite evidence of significant overlap in the incidence of stroke and AD, the impact of the chronic sequelae of stroke on the development and progression of AD-associated pathology is largely unknown. Here, we have developed two novel post-stroke mixed dementia models, which enable the long-term consequences of stroke injury on the development of AD-related pathological markers, such as amyloid and tau to be tracked. Because of the heterogeneity and complexity of human post-stroke mixed dementia [51, 94], each of our models are important for reflecting the varied clinical symptoms and pathological diversity exhibited in mixed dementia patients [42, 44], in which age and a predisposition for, or existing diagnosed or undiagnosed AD symptoms, may affect long-term outcome after a stroke. These models enable hypotheses, such as the myelin repair hypothesis highlighted here to be tested, and provide a platform for future preclinical drug studies.

## Abbreviations

AD: Alzheimer’s disease
wt: Wildtype
Tg: Transgenic
BACE1: β-secretase 1
NGR1: Neuregulin 1
Aβ: β-amyloid
AβPP: Aβ precursor protein
p: Phosphorylated
PS1: Presenilin 1
MCAO: Middle cerebral artery occlusion
PT: Photothrombotic
ET-1: Endothelin-1
LPS: Lipopolysaccharide
mo: Months old
Thy1-hAβPP^Lond/Swe^ (hAPP-SL): Transgenic mouse line 41 over-expresses human AβPP751 containing the London (V717I) and Swedish (K670M/N671L) mutations under the control of the Thy1 promoter
DH stroke: Distal MCAO plus hypoxia
OR: Object relocation
SAB: Spontaneous alternation behavior
NOR: Novel object recognition
FO: Familiar object
LDT: Light dark transition
ChAT: Choline acetyl transferase
GFAP: Glial fibrillary acidic protein
Iba1: Ionized calcium binding adaptor molecule 1
ThioS: Thioflavin S
NeuN: Neuronal specific nuclear protein
epidermal growth factor, EGF: ErbB
ITI: Inter-trial-interval
BBB: Blood brain barrier
